# Identification of organs of origin of macrophages that produce presepsin via neutrophil extracellular trap phagocytosis

**DOI:** 10.1038/s41598-024-66916-y

**Published:** 2024-07-16

**Authors:** Akihiro Kondo, Tatsuya Morinishi, Yusuke Yamaguchi, Akishige Ikegame

**Affiliations:** 1 Laboratory of Hematology, Department of Medical Technology, Kagawa Prefectural University of Health Sciences, 281-1, Hara, Mure-cho, Takamatsu, Kagawa 761-0123 Japan; 2 Laboratory of Pathology, Department of Medical Technology, Kagawa Prefectural University of Health Sciences, Takamatsu, Kagawa Japan

**Keywords:** Presepsin, Macrophage, Neutrophil extracellular traps, Sepsis, Biomarker, Cell biology, Biomarkers

## Abstract

Presepsin (P-SEP) is a specific biomarker for sepsis. Monocytes produce P-SEP by phagocytosing neutrophil extracellular traps (NETs). Herein, we investigated whether M1 macrophages (M1 MΦs) are the primary producers of P-SEP after NET phagocytosis. We co-cultured M1 MΦs and NETs from healthy participants, measured P-SEP levels in the culture medium supernatant, and detected P-SEP using western blotting. When NETs were co-cultured with M1 MΦs, the P-SEP level of the culture supernatant was high. Notably, we demonstrated, for the first time, the intracellular kinetics of P-SEP production by M1 MΦs via NET phagocytosis: M1 MΦs produced P-SEP intracellularly 15 min after NET phagocytosis and then released it extracellularly. In a sepsis mouse model, the blood NET ratio and P-SEP levels, detected using ELISA, were significantly increased (*p* < 0.0001). Intracellular P-SEP analysis via flow cytometry demonstrated that lung, liver, and kidney MΦs produced large amounts of P-SEP. Therefore, we identified these organs as the origin of M1 MΦs that produce P-SEP during sepsis. Our data indicate that the P-SEP level reflects the trend of NETs, suggesting that monitoring P-SEP can be used to both assess NET-induced organ damage in the lungs, liver, and kidneys during sepsis and determine treatment efficacy.

## Introduction

Sepsis is life-threatening organ failure resulting from an inadequately controlled biological response to infection. Sepsis is an important global health problem because it is associated with acute organ dysfunction and a high risk of mortality^1^. Rudd et al. reported that an estimated 48.9 million people worldwide developed sepsis in 2017, and 11 million, or 19.7% of global deaths, were sepsis-related^2^. As early diagnosis of sepsis is critical for the timely initiation of treatment, there is a need for biomarkers that are specifically elevated in the early stages of sepsis and can rapidly assess pathogenesis^3,4^. Presepsin (P-SEP), which is another name for the soluble CD14 subtype, sCD14-ST, is the N-terminal fragment of a cluster of differentiation 14 (CD14), a receptor for lipopolysaccharide (LPS). P-SEP is elevated in the blood of patients with sepsis and is, therefore, a biomarker for this condition^5,6^. CD14, the precursor of P-SEP, is expressed on the plasma membrane of monocytes, macrophages, and neutrophils. When monocytes and neutrophils phagocytose bacteria, they internalize their own CD14, which is degraded by proteolytic enzymes, such as Cathepsin D in lysosomes, and released into the blood as P-SEP with a molecular weight of 13 kDa^7^. Arai et al. stimulated human neutrophils, lymphocytes, and monocytes with bacteria or inflammatory cytokines and reported that P-SEP is secreted by monocytes that phagocytose bacteria^8^. In addition, unlike existing sepsis markers such as procalcitonin, P-SEP does not involve protein synthesis, is elevated in the early stages of infection, and correlates with the severity of sepsis^9–11^. Therefore, P-SEP is a useful marker for rapidly diagnosing and monitoring sepsis severity^12,13^. However, the mechanism of P-SEP production in the body remains to be understood.

In 2004, Brinkmann et al. reported neutrophil extracellular traps (NETs) as one of the biological defense mechanisms of neutrophils^14^. When blood-borne pathogens or cytokines bind to neutrophils, neutrophil nicotinamide adenine dinucleotide phosphate (NADPH) oxidase is activated, and the histones in the neutrophil nucleus are citrullinated. Histone-citrullinated neutrophils release chromatin fibers modified with the intracellular antimicrobial proteins myeloperoxidase and neutrophil elastase. These chromatin fibers released into the blood are NETs^15–17^. NETs released into the blood play an important role in biological defense during sepsis, whereas excessive NETs are involved in developing thrombosis and autoimmune diseases^18–20^.

In our previous study, we confirmed that extracellularly released NETs contain high levels of CD14 and demonstrated that P-SEP is produced as a result of monocytes phagocytosing these NETs^21^. However, monocytes comprise a low percentage of circulating leukocytes in the blood and have a short residence time in the peripheral blood^22^. Therefore, we focused on macrophages (MΦs), which are more abundant than monocytes in each organ, and hypothesized that M1 MΦs, which phagocytose foreign material, are the major P-SEP-producing cells^23,24^. In this study, we tested the hypothesis that M1 MΦs produce P-SEP after NETs phagocytosis using M1 MΦs differentiated from healthy monocytes. We also aimed to identify the organ of origin of M1 MΦs that produce P-SEP using a mouse model of sepsis.

## Results

### M1 MΦs phagocytosing NETs produce P-SEP intracellularly and release P-SEP extracellularly

**Figure 1:**
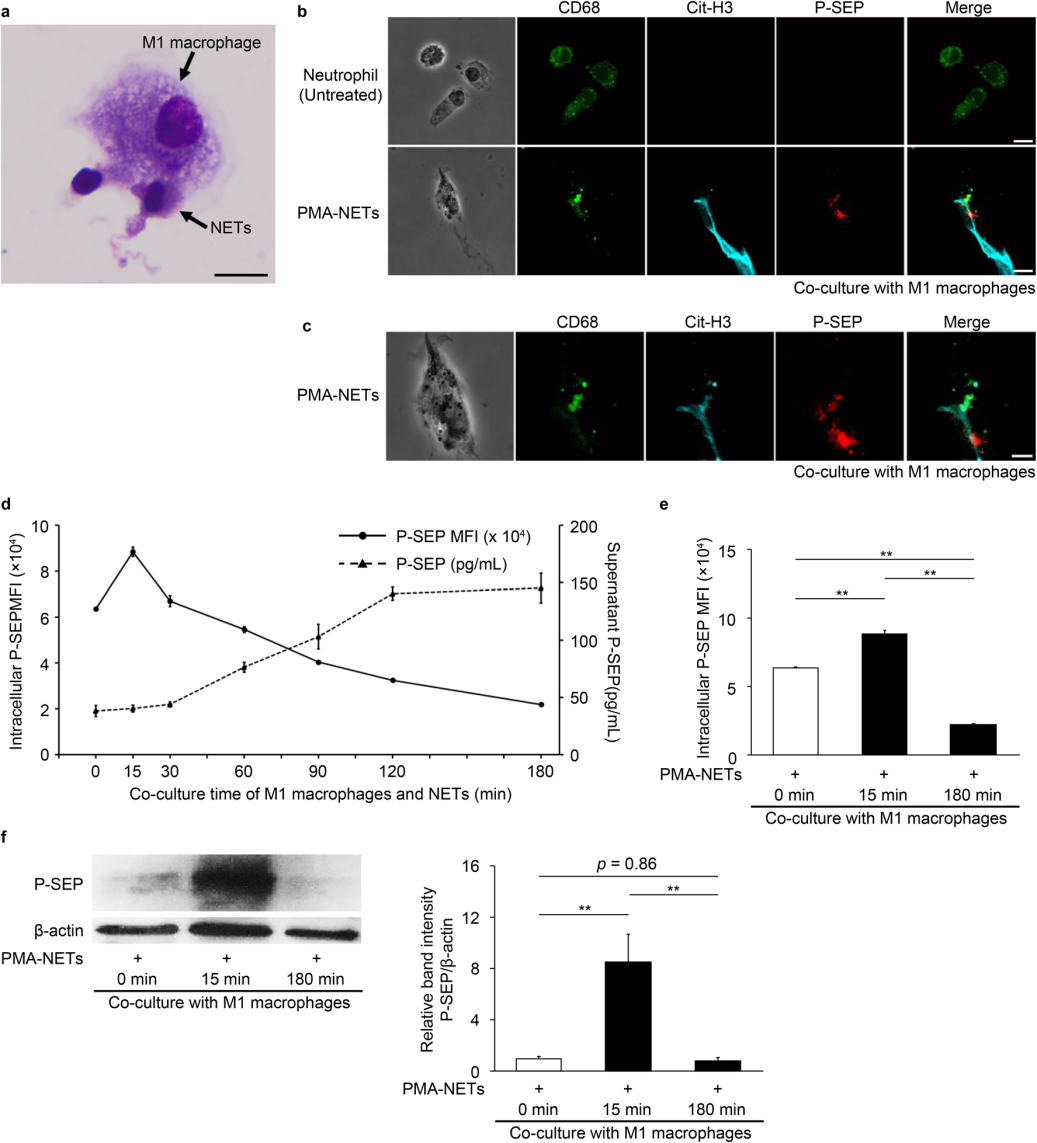
After M1 MΦs phagocytose NETs, P-SEP produced within M1 MΦs is released into extracellular space over time. (**a**) Cell morphology image of M1 MΦ phagocytosing PMA-NETs. (MG stain, magnification 1,000 ×). Scale bar: 10 µm. (**b**) Immunofluorescence staining results of M1 MΦ phagocytosing PMA-NETs. NETs indicated by arrows are citrullinated histone H3-positive. Scale bar: 10 µm. (**c**) Enlarged view of M1 MΦ phagocytosing PMA-NETs. Arrows indicate images of P-SEP production by M1 MΦs phagocytosing NETs. Scale bar: 5 µm. (**d**, **e**) After co-culturing M1 MΦs and NETs, cells were collected over time and analyzed for intracellular P-SEP expression using flow cytometry. P-SEP levels in culture medium supernatant were measured. (**f**) Western blotting of intracellular P-SEP expression intensity and P-SEP at 0, 15, and 180 min after NET phagocytosis by M1 MΦs. P-SEP protein levels were quantified using Image J. β-actin band intensity was used to correct P-SEP band intensity. The unprocessed western blot image is shown in Supplementary Fig. S11. All differences denoted by asterisks were subjected to two-tailed Student’s *t*-tests. Values are presented as mean ± standard deviation (SD; *n* = 3). **p < 0.01. P-SEP, presepsin; NET, neutrophil extracellular trap; M1 MΦs, M1 macrophage.

After the co-culture of M1 MΦs and NETs, the phagocytosis of NETs and dead cells by M1 MΦs was observed, as indicated by the arrows in the photograph shown in Fig. 1a. Immunofluorescent staining also showed that CD68-positive M1 MΦs phagocytosed citrullinated histone H3 (Cit-H3)-positive NETs, and intracellular P-SEP production was observed (Fig. 1b,c).

Using flow cytometry to analyze the intracellular P-SEP expression levels over time in the M1 MΦs phagocytosis of NETs, we observed that the mean fluorescence intensity peaked 15 min after the co-culture and then slowly declined. In contrast, the P-SEP levels in the culture medium supernatant increased slowly after 30 min of co-culturing and peaked at 180 min (Fig. 1d,e). Furthermore, cells were collected after 0, 15, and 180 min of co-culturing with the NETs, and the protein level of P-SEP was detected via western blotting; the highest expression level was observed after 15 min of co-culture (Fig. 1f). These results demonstrated the kinetics of P-SEP production after NET phagocytosis in M1 MΦ.

### P-SEP is produced via M1 MΦ phagocytosis of increased NETs

**Figure 2:**
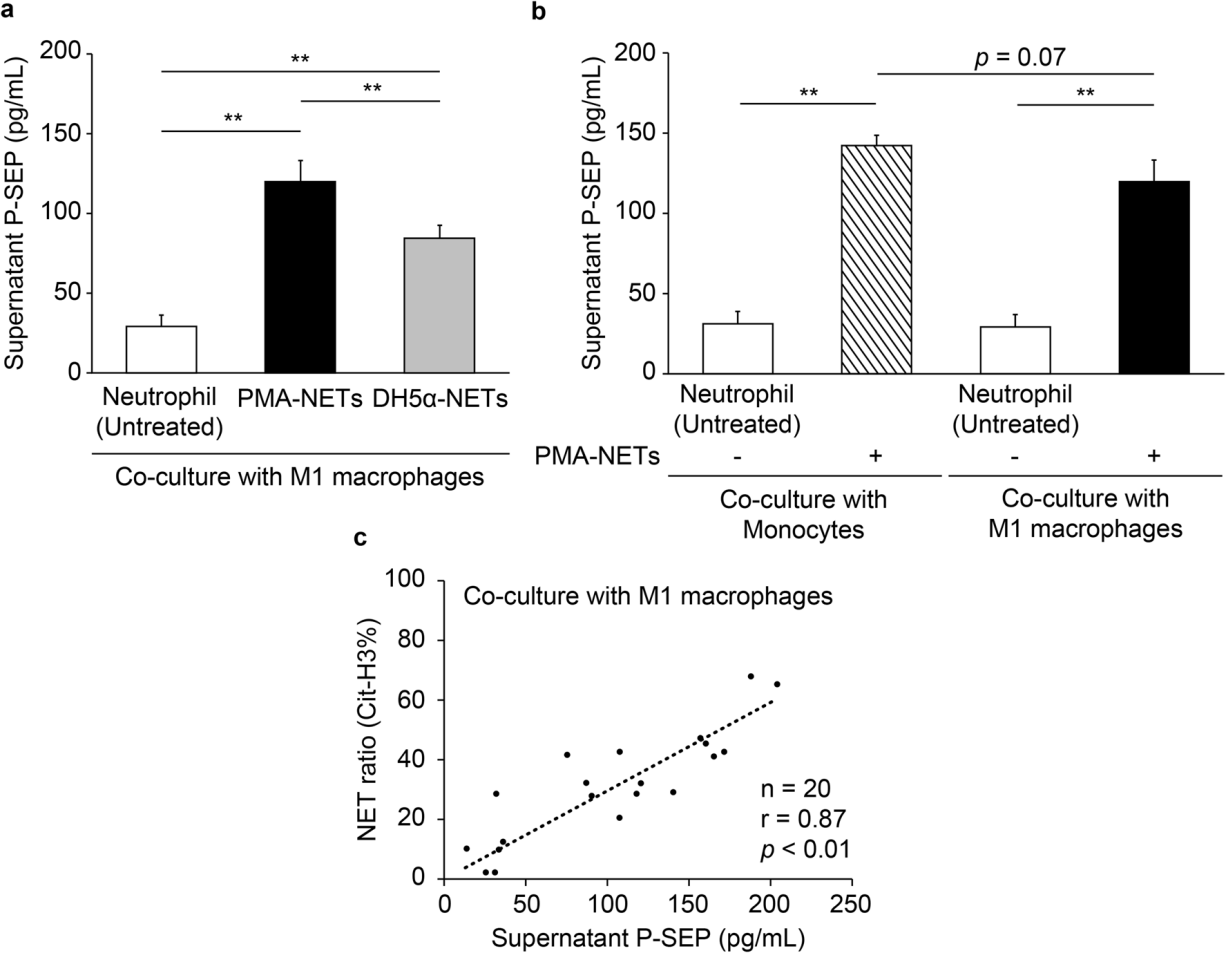
M1 MΦs phagocytose NETs and produce P-SEP. (**a**) P-SEP production after co-culture of M1 MΦs with PMA-NETs and DH5α-NETs was compared. (**b**) P-SEP production after co-culture of M1 MΦs or monocytes with PMA-NETs was compared. (**c**) Correlation between P-SEP production and NET ratio after M1 MΦ phagocytosed NETs. All differences denoted by asterisks were subjected to two-tailed Student’s *t*-tests. Values are presented as mean ± SD (*n* = 3). **p < 0.01. P-SEP, presepsin; NET, neutrophil extracellular trap; M1 MΦs, M1 macrophage.

The P-SEP levels in the culture medium supernatant after the co-culture of M1 MΦs and neutrophils were 29.2 ± 6.0 pg/mL, whereas those after the co-culture with PMA-NETs were 119.7 ± 10.8 pg/mL. P-SEP levels after co-culture with DH5α-NETs were 84.7 ± 6.5 pg/mL, indicating that M1 MΦs phagocytosed the NETs and produced P-SEP (*p* = 0.0005, *p* = 0.005) (Fig. 2a). When monocytes and M1 MΦs from the same individual were co-cultured with NETs, the P-SEP levels were 132.7 ± 3.7 pg/mL after NET phagocytosis by monocytes and 119.7 ± 10.8 pg/mL after NET phagocytosis by M1 MΦs. There was no significant difference in P-SEP levels produced after NET phagocytosis by monocytes and M1 MΦs (*p* = 0.07) (Fig. 2b). Furthermore, a statistically significant correlation was observed between the NET ratio (Cit-H3 positivity) co-cultured with M1 MΦs and the P-SEP level produced by M1 MΦs (r = 0.87, *p* < 0.0001) (Fig. 2c), indicating that P-SEP was produced in proportion to the number of NETs phagocytosed by the M1 MΦs.

### P-SEP production is inhibited by blocking neutrophil NET formation and M1 MΦ phagocytosis

**Figure 3:**
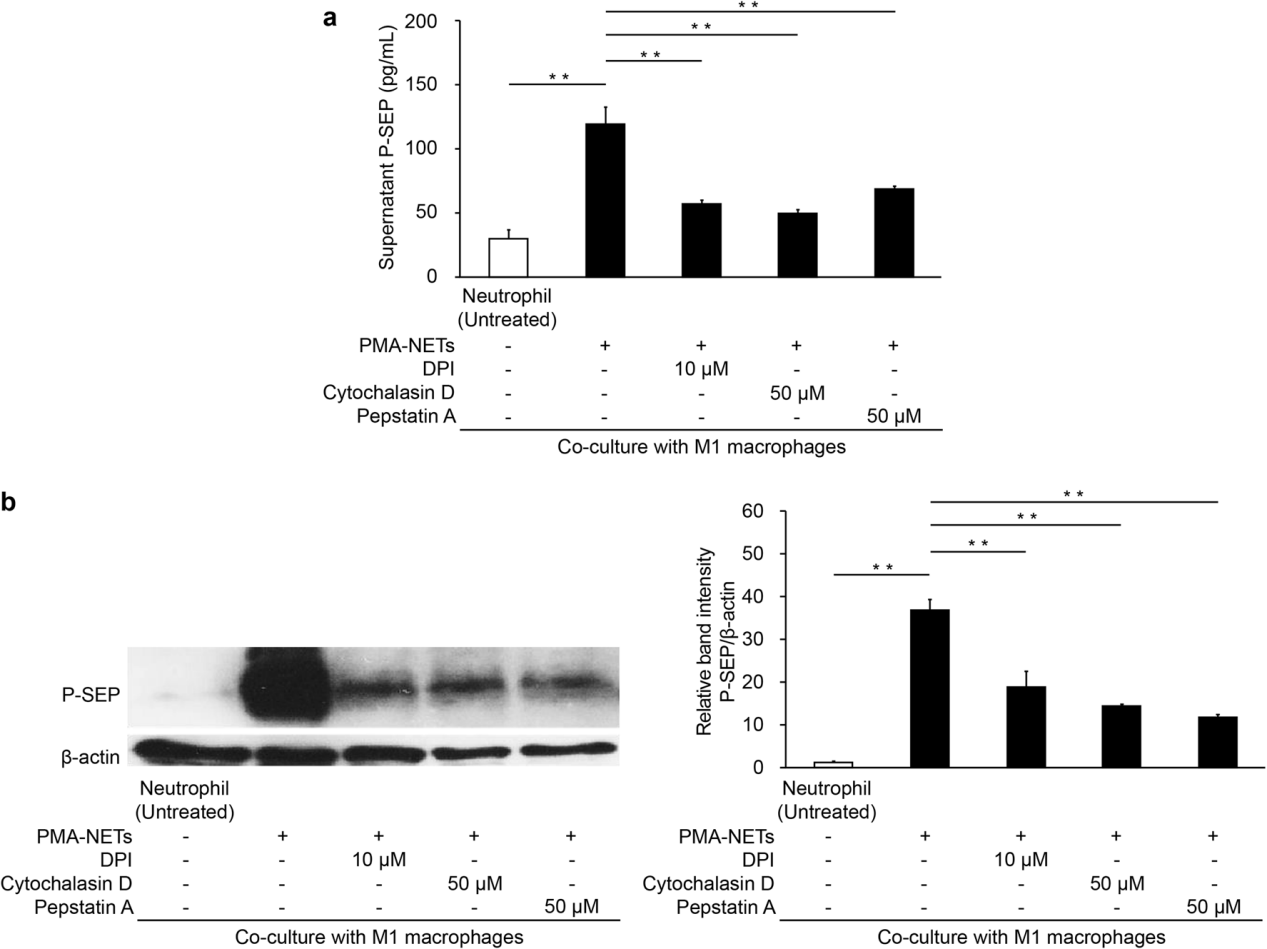
Evaluation of P-SEP levels in inhibiting NET formation, M1 MΦ phagocytosis, and protease inhibition. (**a**) Evaluation of culture medium supernatant P-SEP levels after adding various inhibitors. (**b**) Western blotting of P-SEP protein levels in M1 MΦs after adding various inhibitors. P-SEP protein levels were quantified using Image J. β-actin band intensity was used to correct P-SEP band intensity. The unprocessed western blot image is shown in Supplementary Fig. S12. All differences denoted by asterisks were subjected to two-tailed Student’s *t*-tests. Values are presented as mean ± SD (*n* = 3). **p < 0.01. P-SEP, presepsin; NET, neutrophil extracellular trap; M1 MΦs, M1 macrophage.

The P-SEP levels in the culture medium supernatant after co-culture of M1 MΦs and PMA-NETs were 119.7 ± 10.8 pg/mL. However, the P-SEP levels were significantly low (*p* < 0.0001) at 56.8 ± 2.5 pg/mL after co-culture with neutrophils in which diphenyleneiodonium chloride (DPI) inhibited NET formation. Inhibiting M1 MΦ phagocytosis using Cytochalasin D significantly reduced the P-SEP levels to 48.9 ± 3.1 pg/mL, even after co-culture with NETs (*p* < 0.0001). Furthermore, inhibiting Cathepsin D in M1 MΦs via Pepstatin A significantly reduced P-SEP levels to 68.6 ± 1.6 pg/mL (*p* < 0.0001) (Fig. 3a). Comparing the P-SEP protein levels of M1 MΦs-phagocytosed NETs via western blotting and M1 MΦs after each inhibition assay, the P-SEP protein levels were observed to be significantly decreased after inhibiting NET formation, the phagocytosis of M1 MΦs, and Cathepsin D (*p* < 0.0001, *p* < 0.0001, *p* < 0.0001) (Fig. 3b).

### Mouse models of *sepsis* show elevated P-SEP levels due to increased blood NET ratio

**Figure 4:**
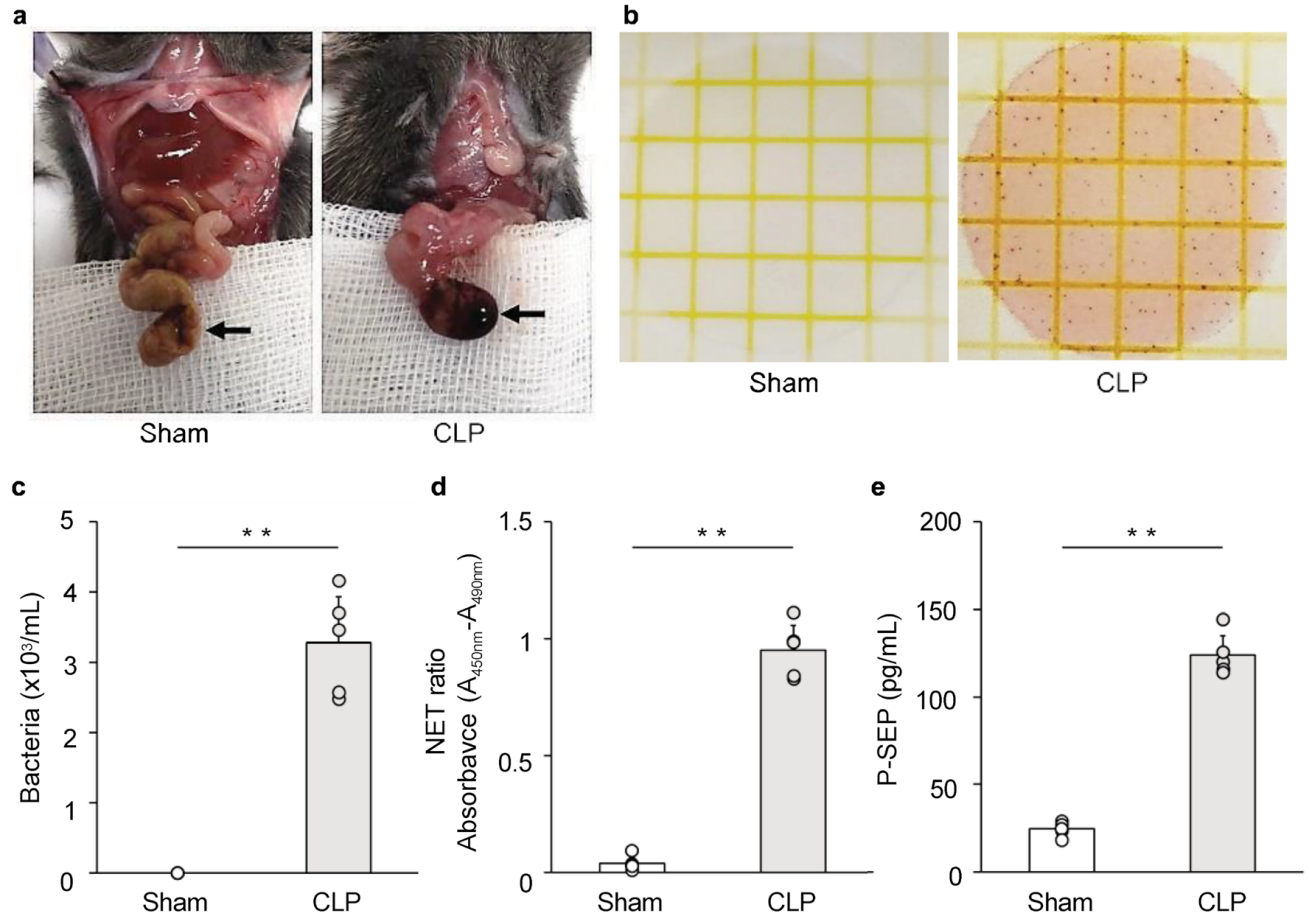
Evaluated blood NET ratio and blood P-SEP production in CLP-treated sepsis model mice. (**a**) Gross cecum findings for the sham group and CLP-treated sepsis model mice. As indicated by arrows, the ceca of CLP-treated mice were necrotic. (**b**, **c**) Comparison of viable bacteria calculated from colony counts. Each dot represents one mouse. (**d**, **e**) Blood NET ratio and P-SEP levels were evaluated in CLP-treated sepsis model mice. Each dot represents one mouse. All differences denoted by asterisks were subjected to two-tailed Student’s *t*-tests. Values are presented as mean ± SD (*n* = 5). **p < 0.01. P-SEP, presepsin; NET, neutrophil extracellular trap; M1 MΦs, M1 macrophage.

Observing the cecum of a mouse model of sepsis 12 h after cecal ligation and puncture (CLP) revealed necrosis of the cecum, as indicated by the arrow in the photograph shown in Fig. 4a. When the mouse serum from each group was cultured, the sham group had 0 measurable enterobacterial colonies on Petrifilm, whereas the sepsis model mice (CLP group) had 163.8 ± 32.5 colonies (Fig. 4b). From the colony count, the number of bacteria in the blood of mice in the CLP group was 3276.0 ± 650.1 bacteria/mL, confirming that the mice in the CLP group developed sepsis (Fig. 4c).

The DNA-histone complexes in the NETs were detected via enzyme-linked immunosorbent assay (ELISA) and evaluated as the blood NET ratio. The NET ratio was significantly higher in the CLP group than in the sham group (Fig. 4d). The plasma P-SEP levels in the sham group were 24.4 ± 3.7 pg/mL, whereas they were significantly higher in the CLP group at 123.9 ± 11.0 pg/mL (*p* < 0.0001) (Fig. 4e).

### MΦs produce P-SEP in the lungs, liver, and kidneys in a mouse model of *sepsis*

**Figure 5:**
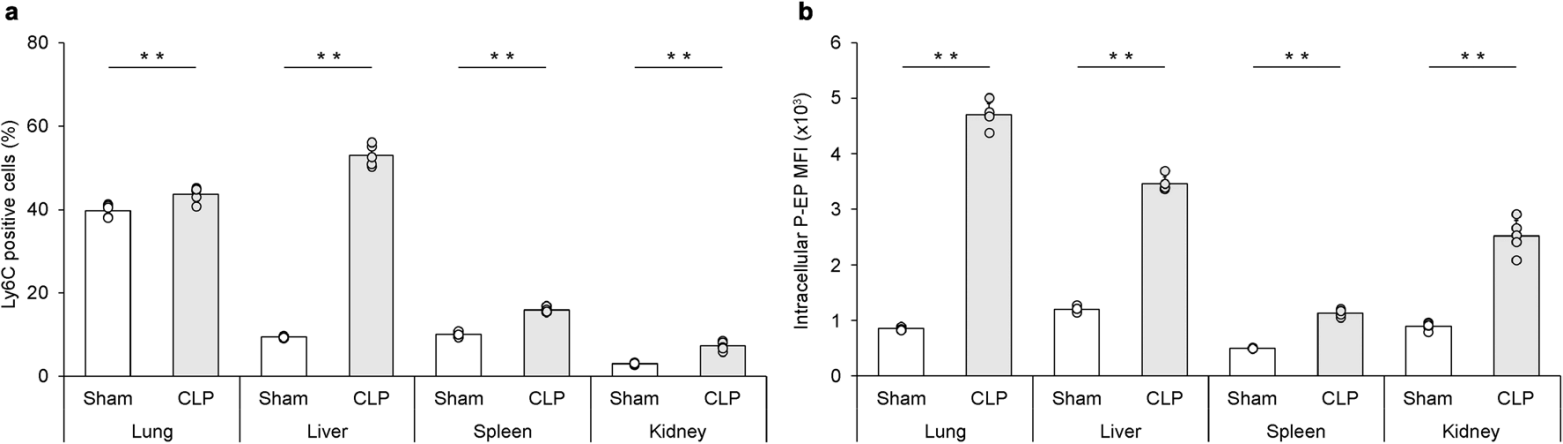
Identification of organs with MΦs producing P-SEP in a mouse model of sepsis. (**a**) Ly6c-positive cells were compared with positivity rate of Ly6C-positive M1 MΦs in each organ as M1 MΦs. M1 MΦs were the most abundant in the lungs of mice in the sham group, with a small number of M1 MΦs in the liver, spleen, and kidneys. Compared to the sham group, mice in the CLP group showed significantly increased Ly6C positivity in each organ, especially markedly in the lungs and liver. Each dot represents one mouse. (**b**) Comparing P-SEP expression in M1 MΦs in each organ between sham and CLP groups, intracellular P-SEP expression in each organ was lower in sham group mice, while P-SEP expression in M1 MΦs in lungs, liver, and spleen was significantly increased in CLP group mice. Each dot represents one mouse. All differences denoted by asterisks were subjected to two-tailed Student’s *t*-tests. Values are presented as mean ± SD (*n* = 5). **p < 0.01. P-SEP, presepsin; M1 MΦs, M1 macrophage.

Analyzing the Ly6C-positive MΦs in mouse organs (lungs, liver, spleen, and kidney) via flow cytometry revealed that in the sham group, the most abundant organ with MΦs was the lungs, with a few MΦs in the liver, spleen, and kidneys. The MΦs in each organ were significantly increased in the CLP group compared to the sham group (*p* = 0.006, *p* < 0.0001, *p* < 0.0001, *p* < 0.0001), especially in the lungs and liver (Fig. 5a). The MΦs in the sham group produced little P-SEP, whereas those in the lungs, liver, and kidneys in the CLP group produced significant amounts of P-SEP (*p* < 0.0001, for all three organs). However, even in the CLP group, the spleen produced less P-SEP than the lungs, liver, and kidneys (Fig. 5b).

**Figure 6:**
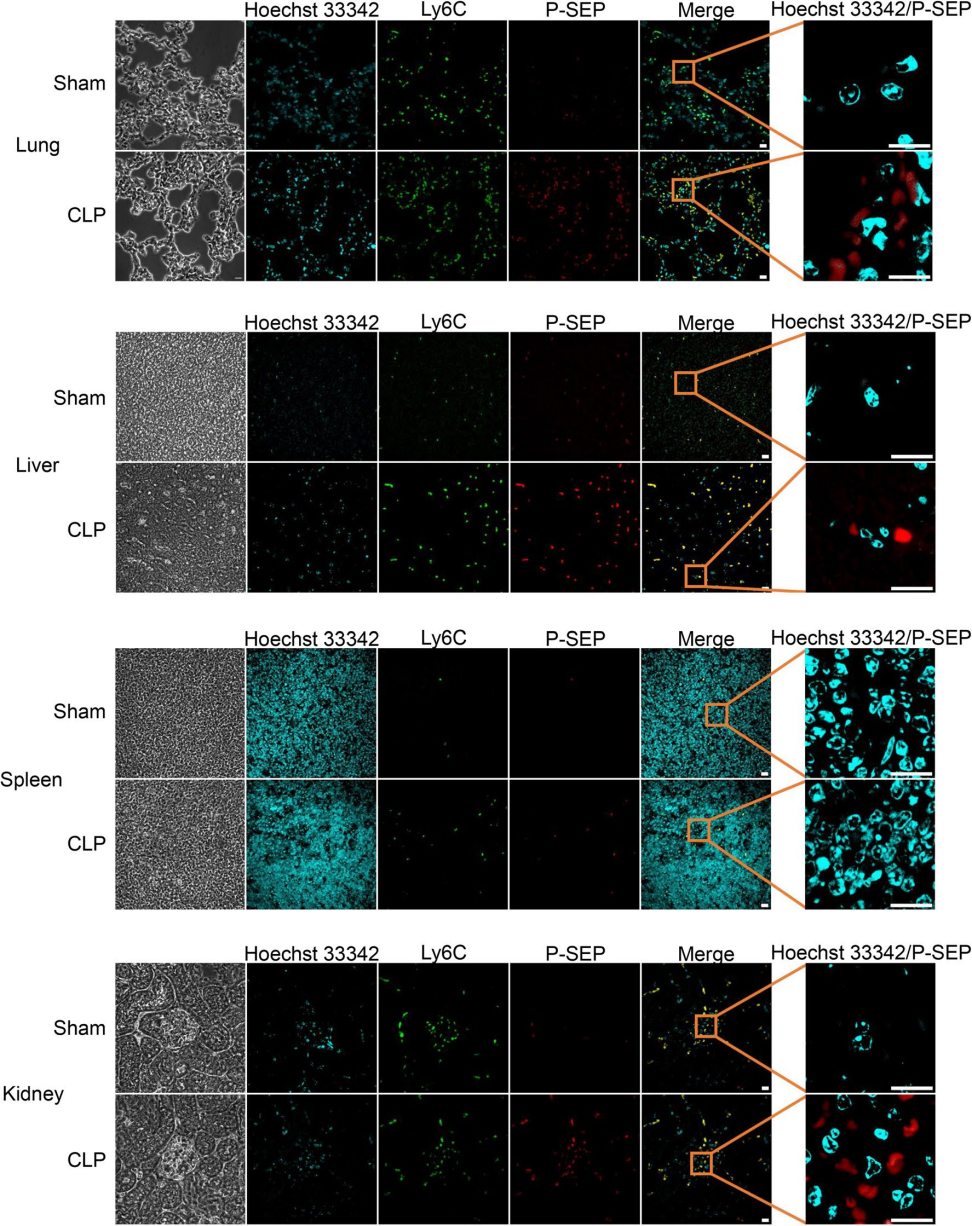
Images of P-SEP production in MΦ of lungs, liver, and kidneys. Immunofluorescence staining for P-SEP in MΦs in each organ showed that only a few MΦs in the sham group were positive for P-SEP, but P-SEP was markedly positive in MΦs from the lungs, liver, and kidney in the CLP group. Scale bar: 10 µm. P-SEP, presepsin; M1 MΦs, M1 macrophage; CLP, cecal ligation and puncture.

Similarly, immunofluorescence staining of each organ revealed few P-SEP-positive MΦs in the sham group, whereas those in the lungs, liver, and kidneys were markedly P-SEP-positive in the CLP group (Fig. 6). Thus, the lungs, liver, and kidneys were identified as the major P-SEP-producing organs.

## Discussion

Elucidating the mechanism of the production of P-SEP, a sepsis biomarker, is important for rapidly diagnosing sepsis. Previously, we demonstrated that P-SEP is produced by monocytes phagocytosing NETs, one of the biological defense mechanisms against infectious bacteria in the blood. However, as monocytes remain in the peripheral blood for a short time, it is likely that the MΦs from which monocytes migrate to the tissues are the major P-SEP-producing cells. In the present study, we determined that MΦs phagocytose NETs and produce P-SEP during sepsis and identified the organ of origin of M1 MΦs.

First, regarding the expression of CD14, the precursor of P-SEP, Chen et al. reported in their study of patients with sepsis and mouse models that the activation of Toll-like receptors on the surface of neutrophils increased CD14 expression and strongly induced inflammatory responses^25^. In our study, we also observed that the NETs induced by DH5α and PMA stimulation contained higher levels of CD14 than the untreated neutrophils did (Supplementary Fig. S1). Consequently, more P-SEP may be produced because M1 MΦs phagocytose these NETs containing high levels of CD14. Conventional methods for measuring NETs using flow cytometry measure the positivity rate by staining extracellularly released nucleic acids with SYTOX Green^26,27^. However, because SYTOX Green stains nucleic acids, it also detects apoptotic cells other than NETs. Therefore, we focused on Cit-H3, which is essential for NET formation.

The NET ratio was more accurately determined by measuring the positivity rate of Cit-H3 released from neutrophils via flow cytometry. A significant correlation was found between Cit-H3 positivity and SYTOX Green positivity (*p* = 0.0004), but Cit-H3 positivity in NET areas was higher than SYTOX Green positivity because it specifically reflects the NET ratio. Analysis of Cit-H3 protein levels via western blotting showed that Cit-H3 protein expression levels were higher in PMA-NETs than in DH5α-NETs (Supplementary Fig. S2). When protein kinase C activated by PMA acts on flavocytochrome b558, which is the catalytic component of NADPH oxidase, NETs are formed to trigger the production of reactive oxygen species from NADPH oxidase^28^. PMA stimulation induces NETs at a higher rate because it directly activates NADPH oxidase without mediating receptors on the plasma membrane. In addition, the expression of flavocytochrome b558 on neutrophil membranes upstream of NET formation was analyzed via flow cytometry, and the expression rate of flavocytochrome b558 was higher when stimulated with PMA than with DH5α. Flavocytochrome b558 expression rate increased in a PMA concentration-dependent manner, suggesting that PMA stimulation specifically induces NET formation and that neutrophil surface Cit-H3 positivity accurately reflects the NET ratio (Supplementary Fig. S2).

MΦs in the organs polarize under the influence of various inducers into M1 MΦs, which phagocytose foreign material and present antigens, and M2 MΦs, which suppress immune responses^29–31^. We hypothesized that the major cells that phagocytose NETs and produce P-SEP are not monocytes that remain in the peripheral blood for short periods of time but instead, M1 MΦs that have migrated from these monocytes to various organs. In this study, by co-culturing M1 MΦs from healthy individuals with NETs derived from neutrophils of the same individuals, we showed that the M1 MΦs phagocytose NETs and produce P-SEP. Zou et al. reported that in a mouse model of sepsis, the blood levels of P-SEP increased after 120 min of infection, peaked at 180 min, and declined at 240–480 min^32^. Our in vitro results also showed that the M1 MΦ produced P-SEP intracellularly 15 min after the phagocytosis of NETs and that the extracellular release of P-SEP peaked at 180 min. This result is also consistent with the timing of increased plasma P-SEP levels in patients with sepsis, suggesting that in vitro experiments reflect P-SEP production in the clinical setting. In summary, to the best of our knowledge, we reported the detailed kinetics of P-SEP production via NET phagocytosis in M1 MΦ for the first time. Furthermore, the short half-life of P-SEP (240 min) and the short duration of P-SEP production in M1 MΦ demonstrated that P-SEP is useful for the early diagnosis of sepsis.

In this study, regardless of the method used to induce NETs, P-SEP levels in the culture medium supernatant were higher after the co-culture of M1 MΦ and NETs. A statistically significant correlation between NET ratio and P-SEP levels was observed, demonstrating that phagocytosis of NETs is largely responsible for P-SEP production (*p* = 0.0004). Furthermore, there was no statistically significant difference in P-SEP production after individual monocytes and M1 MΦ phagocytosed NETs (*p* = 0.07). This result suggests that monocytes and M1 MΦ have similar P-SEP production capacities and that monocytes phagocytose NETs and produce P-SEP while still present in the blood. However, M1 MΦ, which are more abundant than monocytes in various body organs, may be primarily responsible for P-SEP production during sepsis. To confirm that M1 MΦs produce P-SEP via NETs phagocytosis, we performed NET formation inhibition and M1 MΦ phagocytosis inhibition studies. Inhibition studies using DPI, an inhibitor of NET formation, showed that NET formation was inhibited in a DPI concentration-dependent manner, and P-SEP levels of M1 MΦs after NETs phagocytosis were also reduced (Supplementary Fig. S3). In the phagocytosis inhibition assay of M1 MΦs via the addition of Cytochalasin D, inhibition of the phagocytosis of M1 MΦs resulted in a decrease in P-SEP production, indicating that M1 MΦs produce P-SEP via NET phagocytosis. To further demonstrate that Cathepsin D in M1 MΦ degrades internalized CD14 to generate P-SEP, we evaluated P-SEP levels via Cathepsin D inhibition assay using Pepstatin A. Phagocytosis inhibition and Cathepsin D inhibition assays in M1 MΦs showed a concentration-dependent suppression of P-SEP production (Supplementary Figs. S4 and S5). Therefore, we conclude that after M1 MΦs phagocytose NETs containing high levels of CD14, the internalized CD14 is degraded by Cathepsin D to produce P-SEP.

**Figure 7:**
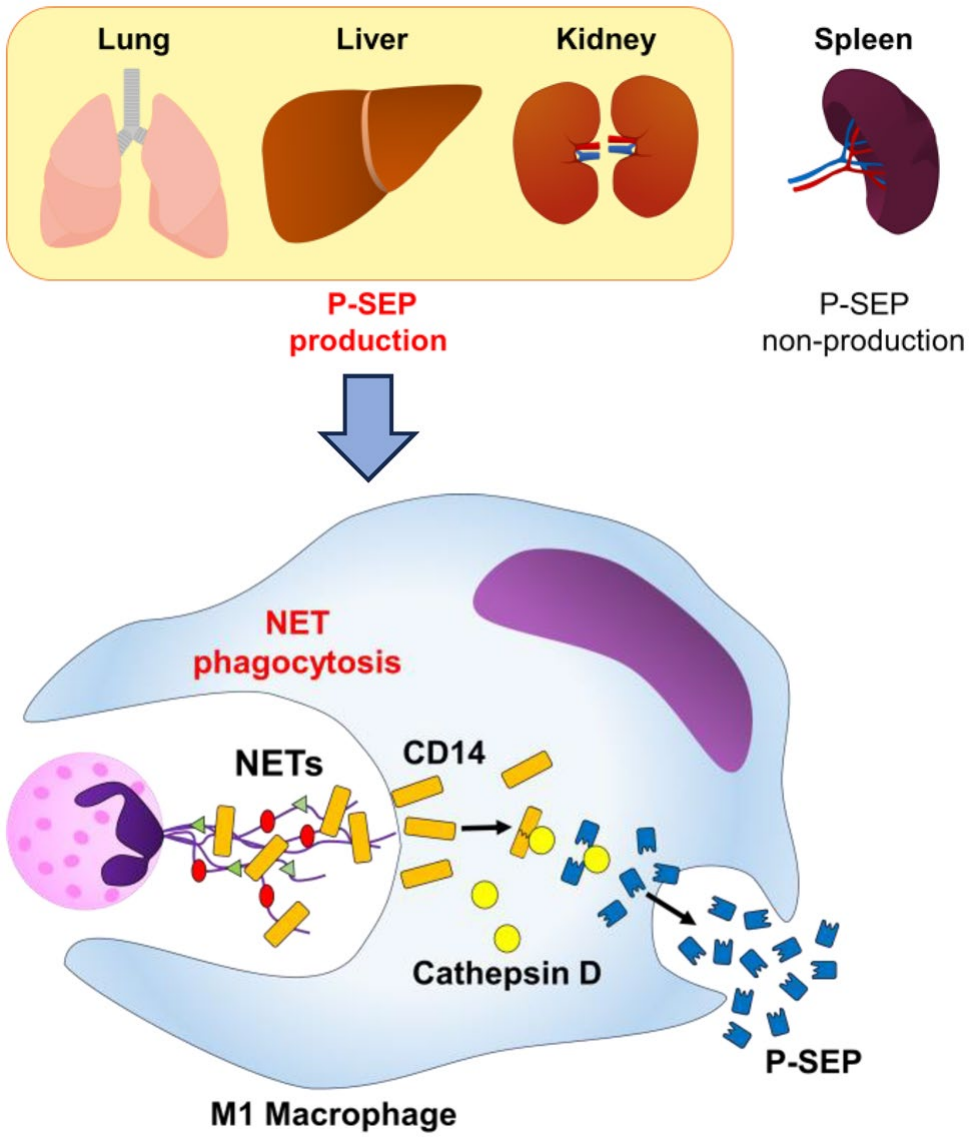
Schematic diagram of the mechanism by which tissue MΦ produces P-SEP to phagocytose NETs. When bacteria infect blood vessels, neutrophils release NETs for biological defense. When MΦs in the lungs, liver, and spleen phagocytose increase NETs in the body, P-SEP is produced, and blood P-SEP levels are high. P-SEP, presepsin; NET, neutrophil extracellular trap; M1 MΦs, M1 macrophage.

The function of MΦs varies depending on the organ in which they are present^33–35^. We focused specifically on MΦs in the lungs, liver, and spleen, which are involved in the removal of dead cells^36^. Urinary P-SEP concentrations are elevated in patients with chronic renal failure, suggesting that renal M1 MΦs may also be involved in P-SEP production^37^. Therefore, in this study, we evaluated the organs of origin of P-SEP-producing M1 MΦs by comparing P-SEP production of MΦs in the lungs, liver, spleen, and kidney of sepsis model mice. In vivo experiments confirmed that sepsis was induced by peritonitis, as intestinal bacteria were significantly increased in the blood of mice 12 h after CLP (*p* < 0.0001). The NET ratio and P-SEP levels in the blood of mice in the CLP group that developed sepsis were significantly higher than those in the sham group (*p* < 0.0001, *p* < 0.0001), and a correlation was observed between the NET ratio and P-SEP levels in vivo. When intracellular P-SEP production was evaluated by flow cytometry, the amount of P-SEP produced by MΦs in the sham group was low. In contrast, in the CLP group, the levels of MΦ increased in each organ, and P-SEP production increased with the onset of sepsis. An in vivo comparison of P-SEP production in each organ in the CLP group revealed that P-SEP production was markedly increased in the lungs, liver, and kidneys, indicating that during sepsis, monocytes migrating from the peripheral blood to these organs differentiate into M1 MΦ and produce P-SEP via NET phagocytosis. Even in the CLP group, the spleen produced less P-SEP than the lungs, liver, and kidneys, identifying these organs as the origins of P-SEP-producing M1 MΦ (Fig. 7). NETs released from neutrophils play a central role in innate immunity by killing pathogens, but increased NETs can cause tissue damage and organ dysfunction, contributing substantially to disease pathogenesis^38,39^. In addition, NETs have been reported to convert tissue MΦs to M1 MΦs, promoting inflammation and exacerbating organ damage^40,41^. These reports suggest that NETs formed in the body of patients with sepsis lead to an increase in M1 MΦs in the lungs, liver, and kidneys. Identifying these organs as the origins of P-SEP-producing M1 MΦ indicates that P-SEP measurement may be useful in assessing damage in these organs caused by NETs during sepsis and may help in determining therapeutic efficacy.

In conclusion, we demonstrated that M1 MΦs produce P-SEP via NET phagocytosis and clarified the previously uncharacterized kinetics of P-SEP production in M1 MΦ. Although there are numerous reports on the association between NETs and organ damage, it is difficult to detect NETs processed by M1 MΦ in each organ, and there are no reports using the NET ratio to assess disease pathogenesis. The detailed cytological mechanism of P-SEP production elucidated in this study indicates that the amount of P-SEP reflects the trend of the NETs, and monitoring measurements using P-SEP, which is easily measured, may be applied in the future to understand the pathogenesis of diseases with increased NETs. Clinical studies examining NETs and P-SEP trends during the treatment of sepsis are needed to provide further assurance.

## Methods

### Reagents and antibodies

Details of reagents used in this study and their sources are as follows: Polymorphprep (Cat. No. 1114683; Axis-Shield, Dundee, Scotland), Phorbol 12-myristate 13-acetate (PMA; Cat. No. P8139; Sigma-Aldrich, St. Louis, MO, USA), *Escherichia coli* DH5α competent cells (DH5α; Cat. No.9057; Takara Bio, Shiga, Japan), SYTOX Green (Cat. No. S7020; Invitrogen, Waltham, MA, USA), Hoechst 33,342 (Cat. No. 346-07,951; Fujifilm, Tokyo, Japan), EasySep Human Monocyte Isolation Kit (Cat. No. 19359; STEMCELL Technologies, Vancouver, BC, Canada), CellXVivo Human M1 Macrophage Differentiation Kit (Cat. No. CDK012; R&D systems, Minneapolis, MN, USA), IntraPrep (Cat. No. A07803; Beckman Coulter, Brea, CA, USA), DPI (Cat. No.81050; Cayman Chemical, Ann Arbor, MI, USA), Cytochalasin D (Cat. No.11330; Cayman Chemical), Pepstatin A (Cat. No. S7381; Selleck Biotech, Houston, TX, USA), May–Grünwald’s stain solution (Cat. No.15053; Mutokagaku, Tokyo, Japan), Giemsa stain solution (Cat. No.15002; Mutokagaku), 1/15 M phosphate buffer solution (pH 6.4) (Cat. No.15612; Mutokagaku), 4% paraformaldehyde phosphate buffer solution (Cat. No.09154-85; Nacalai Tesque, Kyoto, Japan), Skim milk powder (Cat. No. 190-12,865; Fujifilm Wako Pure Chemical, Tokyo, Japan), Chemi-Luna One Ultra (Cat. No. 11644; Nacalai Tesque), Medetomidine hydrochloride (Domitol, ZENOAQ, Fukushima, Japan), Midazolam (Dormicum, Maruishi Pharmaceutical, Osaka, Japan), Butorphanol (Vetorphale, Meiji Seika Pharma, Tokyo, Japan), Cell Death Detection ELISAPLUS (Cat. No. 11774425001; Roche Diagnostics, Basel, Switzerland), and mouse presepsin ELISA Kit (PHC Holdings, Tokyo, Japan).

Details of the antibodies used in this study and their sources are listed in Table 1.

**Table 1:** Details of the antibodies used in this study.

Anti-human myeloperoxidase mouse monoclonal antibody	Cat. No. sc-52707; Santa Cruz Biotechnology, Dallas, TX, USA
Anti-human neutrophil elastase mouse monoclonal antibody	Cat. No. sc-55549; Santa Cruz Biotechnology, Dallas, TX, USA
Anti-human citrullinated histone H3 rabbit polyclonal antibody	Cat. No. ab5103; Abcam, Cambridge, UK
Anti-human presepsin mouse monoclonal antibody (F1106-13–3)	Mochida Pharmaceutical, Tokyo, Japan
Anti-human presepsin rabbit monoclonal antibody (S68)	Mochida Pharmaceutical, Tokyo, Japan
Anti-β-actin rabbit monoclonal antibody	Cat. No. 4970; Cell Signaling Technology, Danvers, MA, USA
Alexa Flour 405-labeled goat anti-rabbit IgG	Cat. No. ab175652; Invitrogen, Waltham, MA, USA
TRITC-labeled goat anti-mouse IgG	Cat. No. SA00007-1; Cosmo Bio, Tokyo, Japan
TRITC-labeled goat anti-rabbit IgG	Cat. No. SA00007-2, Cosmo Bio, Tokyo, Japan
Horseradish peroxidase (HRP)-labeled anti-rabbit IgG	Cat. No. 7074; Cell Signaling Technology, Danvers, MA, USA
FITC-labeled anti-human CD14 mouse monoclonal antibody	Cat. No. 6603511; Beckman Coulter, Brea, CA, USA
PE/Cyanine7-labeled anti-human CD14 mouse monoclonal antibody	Cat. No. 367111; BioLegend, San Diego, CA, USA
APC-labeled anti-human CD45 mouse monoclonal antibody	Cat. No. IM2473; Beckman Coulter, Brea, CA, USA
APC/Fire™ 750-labeled anti-human CD68 mouse monoclonal antibody	Cat. No. 333823; BioLegend, San Diego, CA, USA
PE-labeled anti-human CD80 mouse monoclonal antibody	Cat. No. 560925; BD Biosciences, Franklin Lakes, NJ, USA
PE-labeled anti-human CD86 mouse monoclonal antibody	Cat. No. IM2729U; Beckman Coulter, Brea, CA, USA
FITC-labeled anti-human CD163 mouse monoclonal antibody	Cat. No. 563697; BD Biosciences, Franklin Lakes, NJ, USA
FITC-labeled anti-mouse Ly6C rat monoclonal antibody	Cat. No. 563697; BD Biosciences, Franklin Lakes, NJ, USA
Anti-mouse presepsin rabbit monoclonal antibody (C-pep8)	PHC Holdings, Tokyo, Japan

*CD14* cluster of differentiation 14.

### Ethics approval

This study was conducted per the principles of the Declaration of Helsinki and was approved by the Ethical Review Committee of Kagawa Prefectural University of Health Sciences (Approval No. 404). Written informed consent was obtained from all participants for blood collection and subsequent analyses.

### Experimental animals

Male C57BL/6JJcl mice (age 7 − 9 weeks), free of pathogens, were obtained from CLEA Japan Inc. (Tokyo, Japan). Mice were housed in a specific pathogen-free facility with a 12-h light/dark cycle and free access to food and water. The Animal Experiment Committee of Kagawa Prefectural University of Health Sciences approved the animal experiments (Approval No. 04-6). This study was performed according to ARRIVE guidelines, and all experimental procedures were conducted in accordance with international guidelines for the care and use of laboratory animals.

### Purification of human neutrophils

Peripheral blood was collected from healthy participants in EDTA-2 K blood collection tubes. Anticoagulated peripheral blood was stratified on Polymorphprep. After gradient centrifugation at 500×*g* for 30 min at 20 ± 2 °C, the neutrophil-rich bottom layer was collected. Serum-free RPMI 1640 medium was then added. The samples were washed at 400×*g* for 10 min to obtain purified neutrophils. The purified neutrophils were analyzed using an XS-800i multiparameter automated hematology analyzer (Sysmex, Hyogo, Japan). The purity of the neutrophils was 98.0% or higher.

### Induction of NETs

Purified neutrophils (2.0 × 10^6^ cells/well) suspended in a serum-free RPMI 1640 medium were seeded into 24-well plates (Cat. No. SIAL0526; Sigma-Aldrich). The NETs induced by stimulating neutrophils with 50 nM PMA for 2 h at 37 °C were designated as PMA-NETs. NETs induced by stimulation with DH5α bacterial solution (equivalent to 3.0 × 10^5^ bacteria) adjusted to an optical density of 1.0 for 4 h at 37 °C were designated DH5α-NETs^21^.

### NET ratio measurement using flow cytometry

After NET induction, NET formation was confirmed by staining for Cit-H3, which is essential for NET formation, and measuring the positivity rate via flow cytometry. We also stained extracellularly released nucleic acids with the cell membrane-impermeable DNA stain SYTOX Green^26,27,42^.

After stimulating neutrophils with PMA or DH5α, the cells were collected by pipetting to prepare neutrophil suspensions containing NETs. Anti-Cit-H3 antibody was added to this suspension, incubated for 20 min at room temperature (RT, 20 ± 5 °C), and then incubated with rhodamine (TRITC)-conjugated anti-rabbit immunoglobulin G (IgG) antibody for 20 min at RT. After the antibody reaction, the percentage of Cit-H3 positivity was measured using a CytoFLEX flow cytometer (Beckman Coulter). Similarly, SYTOX Green was added to neutrophil suspensions and incubated for 20 min at RT, and the percentage of SYTOX Green positivity was measured via CytoFLEX (Beckman Coulter). In this study, the NET area was defined as the region containing a cell population with a Cit-H3 positivity greater than 60% on the forward scatter and side scatter (SS) plots of the neutrophils after stimulation with 50 nM PMA for 2 h. The Cit-H3 positivity rate in this NET area was evaluated as the NET ratio^21^ (Supplementary Fig. S6).

### Cit-H3 detection using western blotting

After the induction of NETs by PMA or DH5α, all neutrophil suspensions containing NETs were collected. The suspension was pelleted via centrifugation at 20,000×*g* for 5 min at 4 °C. The pellet was resuspended in 100 µL of SDS sample buffer, boiled for 3 min at 97 °C, and stored at − 80 °C until assayed. Proteins were separated on a 15% polyacrylamide gel and transferred to a PVDF membrane at 120 mA for 60 min. After blocking the membrane with 5% skim milk for 60 min, anti-Cit-H3 antibody (dilution 1:1,000) and anti-β-actin antibody (dilution 1:15,000) were added and incubated for 12 h at 4 °C. After three washes with Tris-buffered saline (TBST) containing 0.05% Tween-20, the cells were incubated with HRP-conjugated anti-rabbit IgG antibody (dilution 1:2,500) for 60 min at RT. After three washes with TBST, Cit-H3 and β-actin bands were detected using Chemi-Luna One Ultra, a chemiluminescence detection reagent for western blotting. Cit-H3 bands were analyzed using Image J software (version 1.8.0) to quantify the Cit-H3 protein levels. β-actin was used as an internal control and corrected by dividing the intensity of the Cit-H3 band by the intensity of the β-actin band^43,44^.

### Isolation of monocytes

EDTA-2 K anticoagulated peripheral blood samples from healthy participants were layered on Polymorphprep, and after gradient centrifugation, the upper layer containing peripheral blood mononuclear cells (PBMCs) was collected. Serum-free RPMI 1640 medium was added, washing twice at 100×*g* for 10 min to remove platelets. The monocytes were then negatively selected from the PBMCs using the EasySep Human Monocyte Isolation Kit. The purified monocytes were analyzed using an XS-800i. The purity of the monocytes was 94.0% or higher.

### Induction of monocyte differentiation into M1 MΦs

A CellXVivo Human M1 Macrophage Differentiation Kit was used to differentiate monocytes into M1 MΦs. The purified monocytes (2.0 × 10^6^ cells/well) were suspended in a medium containing granulocyte macrophage colony-stimulating factor and cultured at 37 °C and 5% CO_2_ for 6 d to differentiate into M1 MΦs. After differentiation, the M1 MΦs were stained with May-Grünwald-Giemsa (Supplementary Fig. S7). The cell morphology was observed at 1,000 × magnification using a BX50 microscope (Olympus, Tokyo, Japan). In addition, CD14, CD45, CD68, CD80, CD86, and CD163 on the cell surface were stained with fluorescently labeled antibodies, and the expression levels of each antigen in CD14 + CD45 + M1 MΦs were analyzed using a CytoFLEX (Beckman Coulter) (Supplementary Figs. S7, S8).

### Evaluation of P-SEP generated by M1 MΦ phagocytosing NETs

The NETs induced via PMA or DH5α stimulation were collected by pipetting to prepare neutrophil suspensions containing NETs. M1 MΦs (5.0 × 10^5^ cells/well) were seeded in 24-well plates and co-cultured with a neutrophil suspension containing NETs from the same individual. The number of neutrophils in the suspension was adjusted to be the same as that in M1 MΦ. After 180 min of co-culture at 37 °C, P-SEP levels in the culture medium supernatant were measured using an automated chemiluminescent enzyme immunoassay, PATHFAST. The controls were co-cultures of M1 MΦs and unstimulated neutrophils (untreated) from the same individual.

### Analysis of P-SEP produced within M1 MΦs phagocytosing NETs

After co-culturing M1 MΦs and NETs, a FITC-conjugated anti-CD14 antibody and APC-conjugated anti-CD45 antibody were added to the cell suspension and incubated for 20 min at RT. After the fixation and permeabilization of the cells using IntraPrep, an anti-P-SEP (S68) antibody was added and incubated for 20 min at RT. TRITC-conjugated anti-rabbit IgG antibody was then added and incubated for 20 min at RT. After washing with phosphate-buffered saline (PBS), the intracellular P-SEP expression levels of CD14-positive M1 MΦs were analyzed using a CytoFLEX (Beckman Coulter).

### Evaluation of P-SEP production kinetics after M1 MΦs phagocytosing NETs

Cell suspensions were collected after 0, 15, 30, 60, 90, 120, and 180 min of co-culture of M1 MΦs and PMA-NETs. The P-SEP production kinetics were evaluated by measuring the P-SEP levels in the culture medium supernatant using a PATHFAST and analyzing the intracellular P-SEP expression levels using a CytoFLEX (Beckman Coulter) (Supplementary Fig. S9).

### P-SEP production imaging in M1 MΦ phagocytosis of NETs using immunofluorescence staining

M1 MΦs (5.0 × 10^5^ cells/well) were seeded on coverslips of 24-well plates and co-cultured with NETs for 60 min at 37 °C. After co-culture, the cells on coverslips were washed with PBS and fixed with 4% paraformaldehyde PBS for 10 min. After washing with PBS, the cells were blocked with 0.1% Triton X-100/PBS containing 5% rat serum for 60 min. The cells were then incubated with an anti-Cit-H3 antibody (dilution 1:1,000) and an anti-P-SEP (F1106-13–3) antibody (dilution 1:2,000) for 60 min at RT. After two washes with PBS, the samples were incubated with Alexa Fluor 405-conjugated anti-rabbit IgG antibody (dilution 1:1,000), TRITC-conjugated anti-mouse IgG antibody (dilution 1:5,000), and FITC-conjugated anti-CD68 antibody (dilution 1:1,000) for 90 min at RT. After two washes with PBS, the coverslips were placed on the glass slides, which were sealed with 80% glycerol and imaged using a FLUOVIEW FV10i confocal microscope (Olympus)^45^.

### Detection of P-SEP produced by M1 MΦs after NET phagocytosis using western blotting

After co-culturing the M1 MΦs and PMA-NETs for 15 min, the cell suspension was collected. The suspension was pelleted via centrifugation at 20,000×*g* for 5 min at 4 °C. The pellet was resuspended in 100 µL of SDS sample buffer, boiled for 3 min at 97 °C, and stored at − 80 °C until assayed. The proteins were separated on a 15% polyacrylamide gel and transferred to a PVDF membrane at 120 mA for 60 min. After blocking the membrane with 5% skim milk for 60 min, anti-P-SEP (S68) antibody (dilution 1:5,000) and anti-β-actin antibody (dilution 1:15,000) were added and incubated for 12 h at 4 °C. After three washes with TBST, the cells were incubated with HRP-conjugated anti-rabbit IgG antibody (dilution 1:2,500) for 60 min at RT. After three washes with TBST, the P-SEP and β-actin bands were detected using Chemi-Luna One Ultra. The P-SEP bands were analyzed using Image J software (version 1.8.0) to quantify the P-SEP protein levels. β-actin was used as an internal control, and protein expression was corrected by dividing the intensity of the P-SEP band by that of the β-actin band.

### Comparison of P-SEP production by monocytes and M1 MΦs phagocytosing NETs

Monocytes and M1 MΦs from the same individual, adjusted to a cell count of 5.0 × 10^5^ cells/well, were each seeded in a 24-well plate and co-cultured with NETs for 180 min at 37 °C. The P-SEP levels in the culture medium supernatant were measured using a PATHFAST and compared with those produced from monocytes and M1 MΦs.

### Effect of inhibitors on P-SEP production in M1 MΦs

Neutrophils and M1 MΦs were preincubated with various inhibitors for the inhibition studies. As controls, M1 MΦs and neutrophils were co-cultured with M1 MΦ without inhibitors. DPI, an inhibitor of NADPH oxidase activity, was used to inhibit NET formation. Purified neutrophils (2.0 × 10^6^ cells/well) were treated with 10 µM DPI for 30 min at 37 °C before PMA stimulation; after NET inhibition, they were co-cultured with M1 MΦs for 180 min at 37 °C. The P-SEP levels in the culture medium supernatant were measured using PATHFAST.

Cytochalasin D was used to inhibit the phagocytosis of M1 MΦs, and Pepstatin A was used to inhibit Cathepsin D. Before the co-culture with NETs, the M1 MΦs (5.0 × 10^5^ cells/well) were treated with 50 µM Cytochalasin D or 50 µM Pepstatin A for 30 min at 37 °C. NETs and M1 MΦs, after inhibitor treatment, were co-cultured for 180 min at 37 °C. The P-SEP levels in the culture medium supernatant were measured using PATHFAST. M1 MΦs and NETs, after adding each inhibitor, were collected 15 min after the co-culture, and P-SEP protein levels were detected via western blotting.

### Generation of sepsis model mice

C57BL/6JJcl mice were subjected to CLP to induce sepsis. Mice were randomly divided into groups of five mice each: the sham operation group (sham group) and the CLP group. Mice were anesthetized via intraperitoneal administration (0.05 mL/10 g) of a mixture of medetomidine hydrochloride (0.3 mg/kg), midazolam (4.0 mg/kg), and butorphanol tartrate (5.0 mg/kg)^46^. A 1-cm midline laparotomy was performed to expose the cecum along the adjacent intestine. The distance from the distal end of the cecum to the ligation point at this time was approximately 1 cm, 60% of the total cecum. The cecum was then gently compressed, and a small amount of stool was extruded from the perforation site. The cecum was returned to its normal intra-abdominal position, and the abdomen closed; the mice in the sham group underwent a similar laparotomy without CLP. The mice were resuscitated via subcutaneous injection of 1 mL pre-warmed saline^47,48^.

The induction of sepsis was confirmed by measuring viable bacterial counts in the mouse serum. Blood was aseptically collected from the tail of anesthetized mice 12 h after CLP, and the serum was separated via centrifugation at 2,000×*g* for 10 min. The serum was diluted 20-fold in sterile PBS and seeded on 3 M™ Petrifilm™ viable bacterial count plates (Cat. No. 6400AC; 3 M, St. Paul, MN, USA). After incubation for 48 h at 35 °C, the number of enterobacterial colonies was counted visually, and the viable count was calculated.

### Evaluation of blood NET ratio and plasma P-SEP levels in sepsis mouse model via ELISA

The anesthetized mice were opened 12 h after CLP, and blood was collected from the heart using a syringe with a 24G needle. The blood obtained was aliquoted into EDTA-2 K collection tubes and centrifuged at 2,000×*g* for 10 min to separate the plasma.

The NET ratio in the mouse blood was assessed using Cell Death Detection ELISAPLUS. Mouse plasma was added to a streptavidin-coated microplate. The DNA-histone complexes in NETs were detected via sandwich ELISA using a biotinylated anti-histone antibody and a peroxidase-conjugated DNA antibody. The absorbance of the substrate at 405 nm in ABTS solution was measured using a microplate reader (Bio-Rad Laboratories, Hercules, CA, USA), and the concentration of DNA-histone complexes released into the mouse plasma was evaluated as the NET ratio^49^.

The P-SEP levels in the mouse plasma were measured using a mouse presepsin ELISA Kit. The P-SEP in the mouse plasma was supplemented with anti-mouse P-SEP antibody solidified in a microplate. The P-SEP was detected via sandwich ELISA using a biotinylated mouse anti-P-SEP antibody. Peroxidase-labeled avidin was then bound to the formed antibody-P-SEP-antibody complex, and the absorbance at 450 nm in tetramethylbenzidine solution was measured using a microplate reader. The absorbances of the samples were plotted on a calibration curve prepared from the measurements of the standards to quantify the mouse plasma P-SEP levels^50^. Each assay was performed with n = 5 mice.

### Analysis of P-SEP in mouse MΦs using flow cytometry

The anesthetized mice were subjected to laparotomy 12 h after CLP, and the organs (lungs, liver, spleen, and kidneys) were harvested. The unfixed organs were unwrapped with a scalpel to disperse the cells, which were washed with PBS and filtered through a 100-µm nylon mesh (Cat, No. 2-9566-05; AZ ONE, Osaka, Japan). Cell suspension solution (100 µL, 1.0 × 10^4^ cells/µL), adjusted to 1.0 × 10^6^ cells/µL with PBS, was then added. FITC-conjugated anti-mouse Ly6C antibody was then added and incubated for 20 min at RT. After the cells were fixed and permeabilized with IntraPrep, an anti-mouse P-SEP (C-pep8) antibody was added and incubated for 20 min at RT. After washing with PBS, the TRITC-conjugated anti-rabbit IgG antibody was added and incubated for 20 min at RT. After staining, the intracellular P-SEP production in mouse M1 MΦs was analyzed using a CytoFLEX (Beckman Coulter). In this study, Ly6C-positive cells in each organ were analyzed as MΦs based on the report that monocyte-derived MΦs express Ly6C^51^ (Supplementary Fig. S10).

### P-SEP production imaging in tissue MΦs using immunofluorescence staining

Anesthetized mice were subjected to laparotomy 12 h after CLP, and the organs (lungs, liver, spleen, and kidneys) were harvested. The organs were fixed in 20% formalin for 24 h at RT and embedded in paraffin. The formalin-fixed paraffin-embedded tissues were cut into 3–5-µm sections. The sections were deparaffined in xylene and rehydrated in ethanol. Antigen retrieval was conducted via autoclave heating for 15 min at 120 ˚C in 0.01 M citrate buffer (pH 6.0) containing 38 mg/dL citric acid monohydrate and 241 mg/dL trisodium citrate dehydrate. After washing with PBS, the cells were blocked with PBS containing 5% rat serum for 60 min. The sections were then incubated with anti-mouse P-SEP antibody (C-pep8, dilution 1:500) and FITC-conjugated anti-Ly6C antibody (dilution 1:1,000) for 60 min at RT. After two washes with PBS, the cells were incubated with TRITC-conjugated anti-rabbit IgG antibody (dilution 1:200) and Hoechst 33,342 (dilution 1:2,000) for 90 min. After two washes with PBS, the sections were sealed with 80% glycerol, and Ly6C-positive MΦs and intracellular P-SEPs were imaged using a FLUOVIEW FV10i (Olympus).

### Statistical analysis

Unless otherwise stated, the data presented were obtained from independent experiments using neutrophils and M1 MΦs isolated from three different blood donors. Each assay was measured once for each blood donor. All statistical analyses were performed using JMP Pro 17 (SAS Institute, Cary, NC, USA). Data are expressed as means ± standard deviation. Student’s *t*-test was used to compare the parastatistical significance between the two groups, and statistical significance was set at *p* ≤ 0.05. In the graphically presented data, * and ** denote *p*-values less than 0.05 and 0.01, respectively.

### Supplementary Information

Supplementary Information.

## Data Availability

The data used and analyzed during the current study are available from the corresponding author upon reasonable request.
