# Supplementary material for: Identification of organs of origin of macrophages that produce presepsin via neutrophil extracellular trap phagocytosis
Source: Sci Rep. 2024 Jul 16;14:16386. doi: 10.1038/s41598-024-66916-y (PMC11252129; doi:10.1038/s41598-024-66916-y)
Supplement: Supplementary file 1 — Supplementary Information. [file 41598_2024_66916_MOESM1_ESM.pdf]

Supplementary information

Identification of organs of origin of macrophages that produce presepsin via neutrophil extracellular trap phagocytosis

Akihiro Kondo[1]*, Tatsuya Morinishi[2], Yusuke Yamaguchi[1], Akishige Ikegame[1]

[1] Laboratory of Hematology, Department of Medical Technology, Kagawa Prefectural University of Health Sciences, Takamatsu, Kagawa, Japan

[2] Laboratory of Pathology, Department of Medical Technology, Kagawa Prefectural University of Health Sciences, Takamatsu, Kagawa, Japan

*Correspondence: Akihiro Kondo, kondou-a@kagawa-puhs.ac.jp

281-1, Hara, Mure-cho, Takamatsu, Kagawa, 761-0123, Japan

Tel: +81-87-870-1212

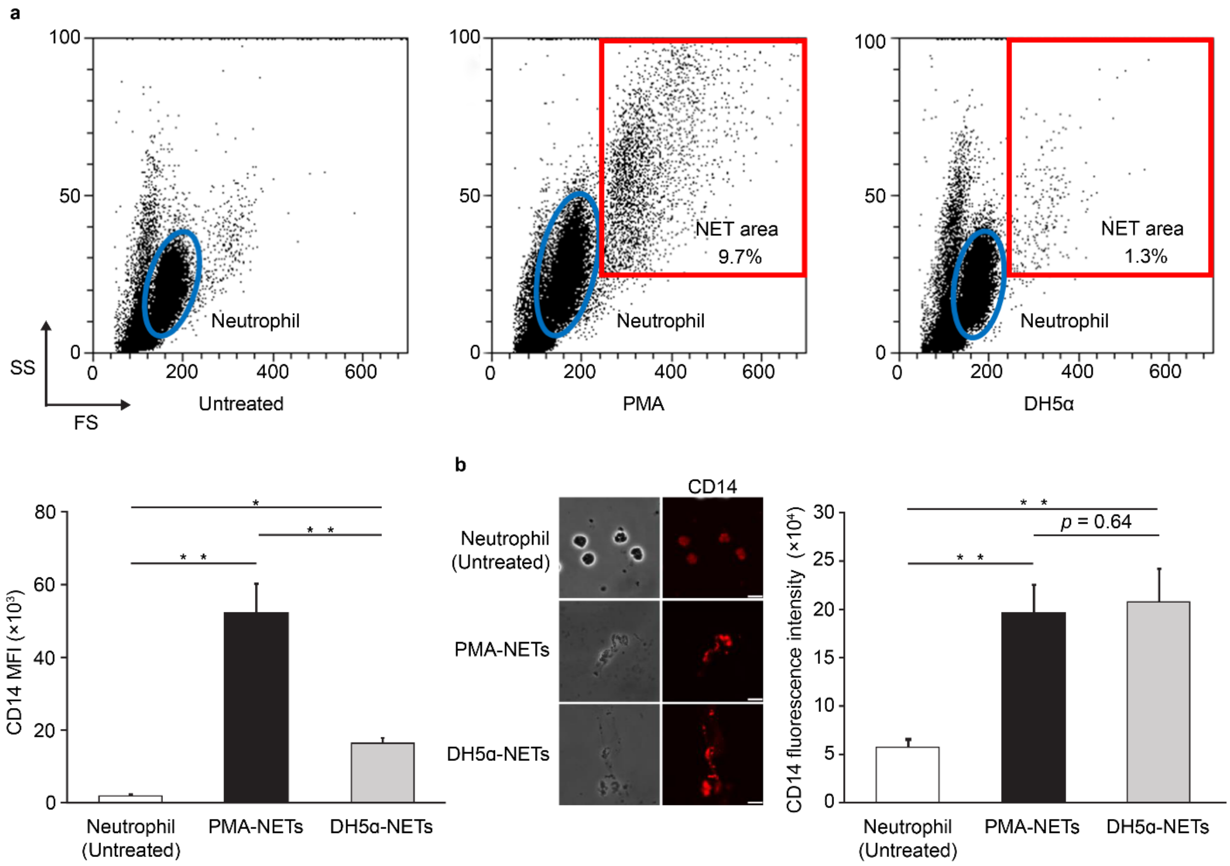

Figure S1. NETs contain high levels of CD14. (a) Expression intensities of CD14 in untreated neutrophils and the NET area were analyzed and compared via flow cytometry. (b) Immunostaining results of untreated neutrophils and NETs. The fluorescence intensity of CD14 was quantified using ImageJ (National Institutes of Health) and compared. NETs induced by PMA and DH5α stimulation contained more CD14 than untreated neutrophils. Scale bar: 10 μm. All differences denoted by asterisks were subjected to two-tailed Student's *t*-tests. Values are presented as mean ± standard deviation (SD; $n = 3$). $^{**}p < 0.01$. $^{*}p < 0.05$.

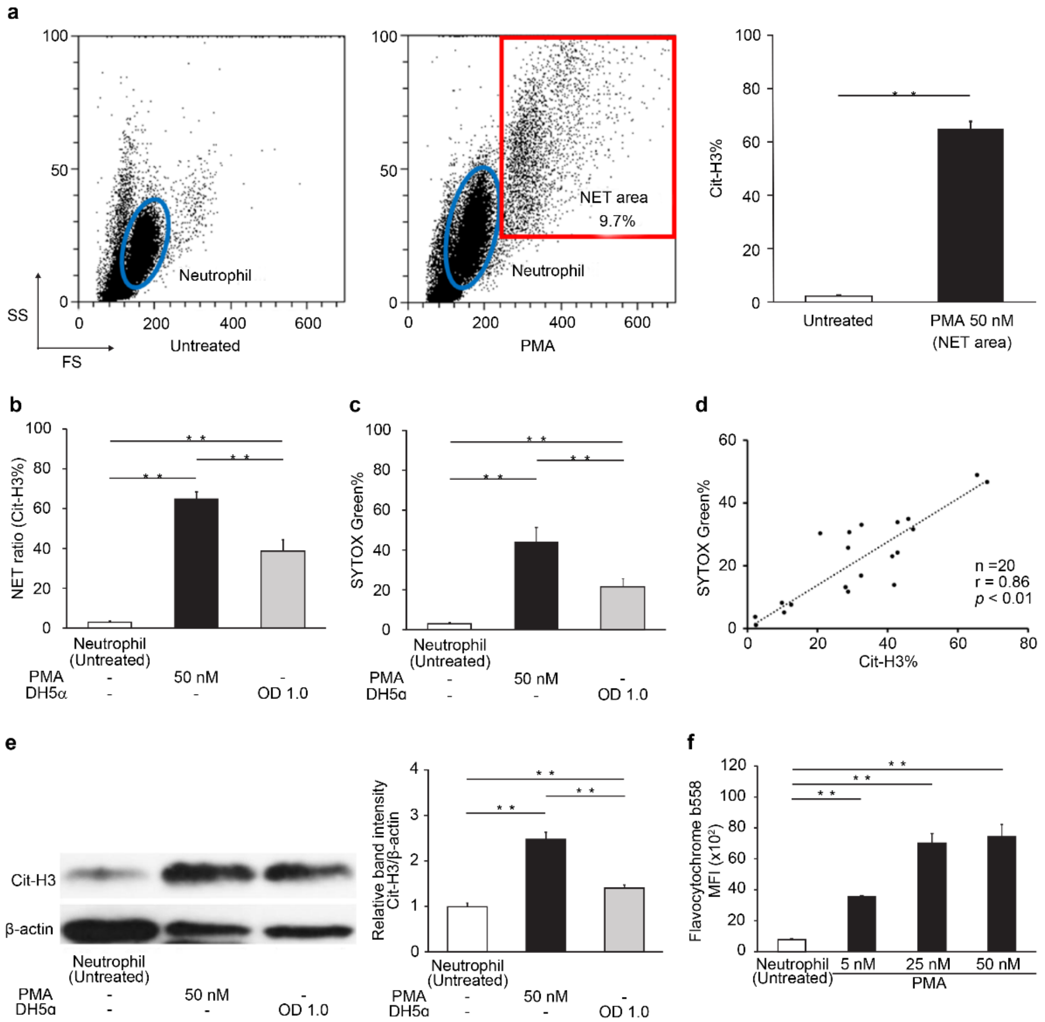

Figure S2. NETs are induced in neutrophils isolated from the peripheral blood of healthy participants by stimulation with PMA and DH5α. (a) Flow cytometry analysis and comparison of Cit-H3 and SYTOX Green positivity rates in untreated neutrophils and NET areas. The NET area was defined as the region containing cell populations with Cit-H3 positivity > 60% in side and forward scatter plots after PMA stimulation. (b, c) NETs were induced in neutrophils with PMA and DH5α, and Cit-H3-positive and SYTOX Green-positive rates were compared. (d) Correlation between Cit-H3-positive rate and SYTOX Green-positive rate in the NET area. (e) Western blotting analysis of Cit-H3 in untreated neutrophils and NETs. Cit-H3 protein levels were quantified using ImageJ (National Institutes of Health), and β-actin band intensity was used to correct Cit-H3 band intensity. The unprocessed western blot image is shown in Supplementary Fig. S13. (f) PMA-stimulated changes in flavocytochrome b558 expression intensity. NETs induced

via PMA stimulation showed enhanced expression of flavocytochrome b558. All differences denoted by asterisks were subjected to two-tailed Student's *t*-tests. Values are presented as mean ± SD ($n = 3$). $^{**}p < 0.01$.

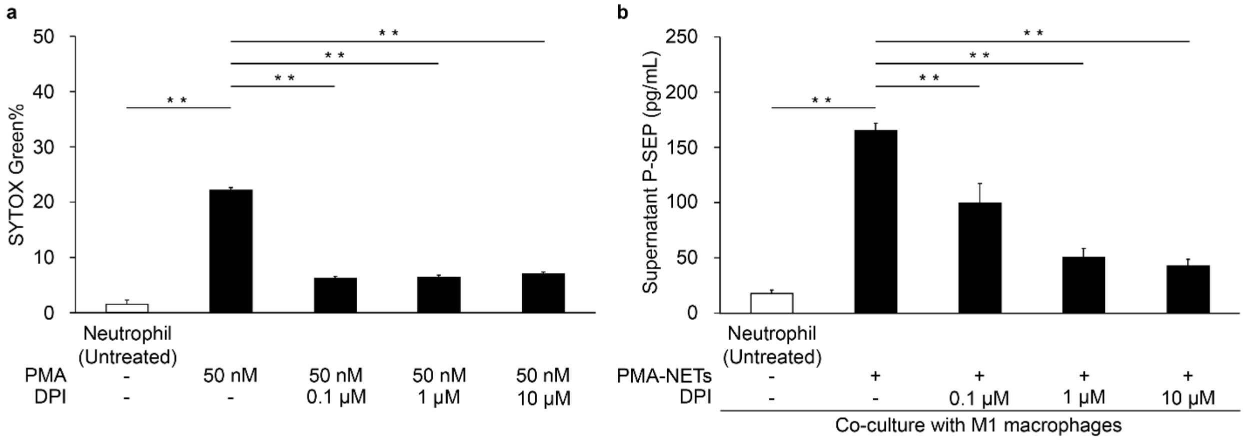

Figure S3. NET formation is suppressed by DPI, which further suppresses P-SEP production in M1 MΦs. (a) NET ratio suppressed by DPI. Neutrophils treated with DPI suppressed NET formation even when stimulated with PMA. Inhibition of NET formation was also dependent on DPI concentration. (b) P-SEP levels in the culture medium supernatant after co-culture of NET-suppressed neutrophils and M1 MΦs. Inhibition of NET formation by DPI also suppressed P-SEP production in co-cultured M1 MΦs. All differences denoted by asterisks were subjected to two-tailed Student's *t*-tests. Values are presented as mean ± SD ($n = 3$). $^{**}p < 0.01$.

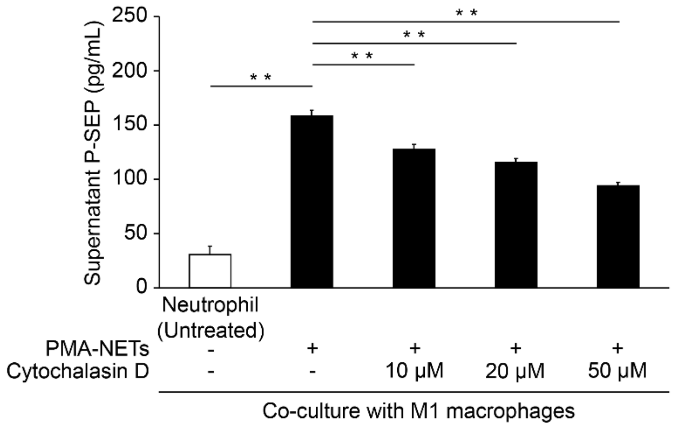

Figure S4. Inhibition of phagocytosis of M1 MΦ by Cytochalasin D suppresses P-SEP production in M1 MΦs. P-SEP levels of culture medium supernatant after co-culturing M1 MΦ and PMA-NETs with phagocytosis suppressed by Cytochalasin D. Inhibition of NET phagocytosis suppressed P-SEP production of M1 MΦs. All differences denoted by asterisks were subjected to two-tailed Student's *t*-tests. Values are presented as mean ± SD ($n = 3$). $^{**}p < 0.01$.

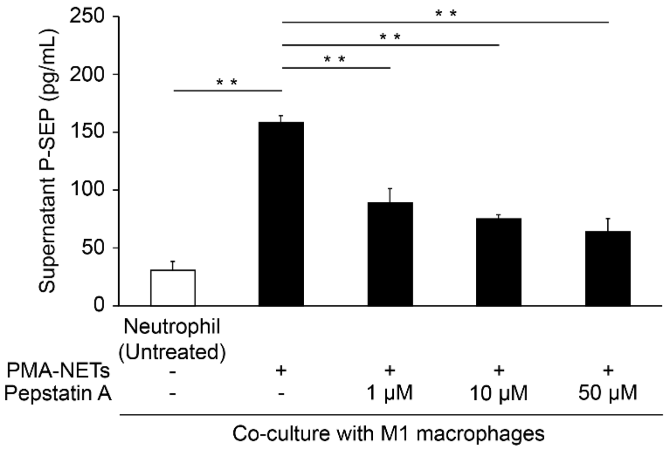

Figure S5. Inhibition of CD14 degradation by Cathepsin D suppresses P-SEP production in M1 MΦs P-SEP levels in the culture medium supernatant after M1 MΦs and PMA-NET co-culture that followed Cathepsin D inhibition by Pepstatin A. Inhibition of CD14 degradation by Pepstatin A suppressed P-SEP production in M1 MΦs. All differences denoted by asterisks were subjected to two-tailed Student's *t*-tests. Values are presented as mean ± SD ($n = 3$). $^{**}p < 0.01$.

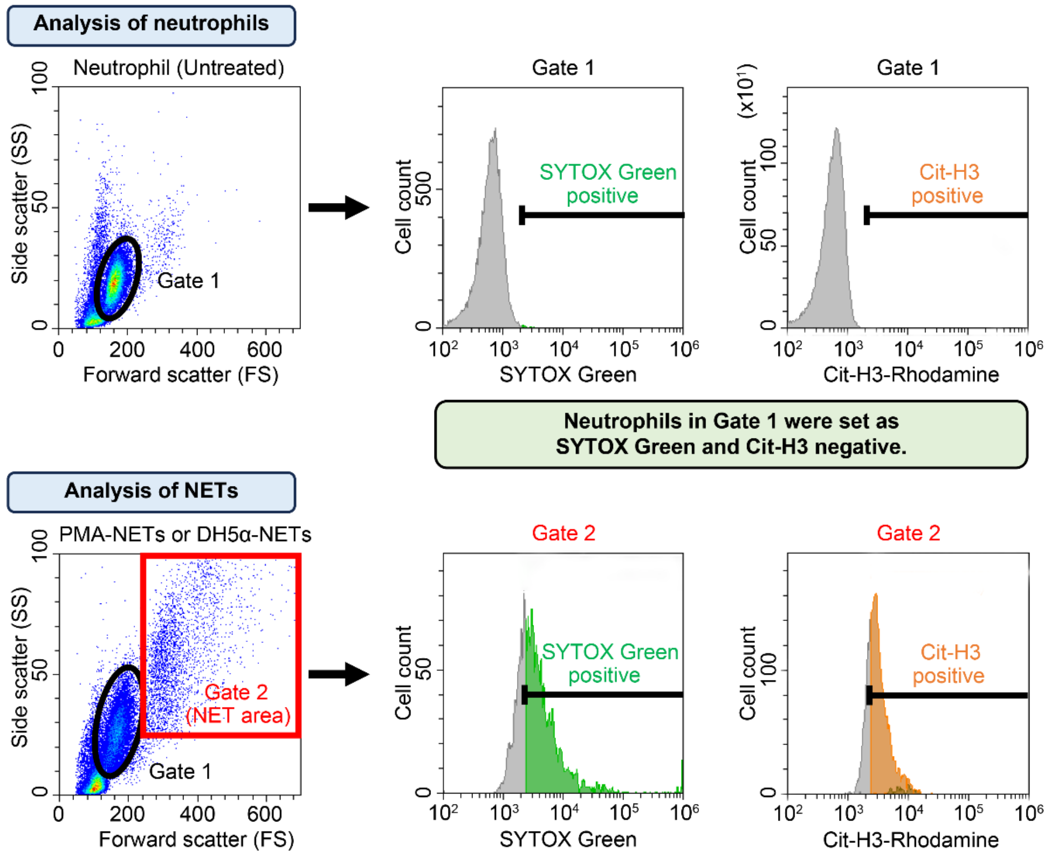

Figure S6. Gating strategy for evaluating the NET ratio using flow cytometry. Neutrophils were set as SYTOX Green and Cit-H3 negative. SYTOX Green-positive rate and Cit-H3-positive rate in the NET area were measured. Cit-H3-positive rate in the NET area was evaluated as the NET ratio.

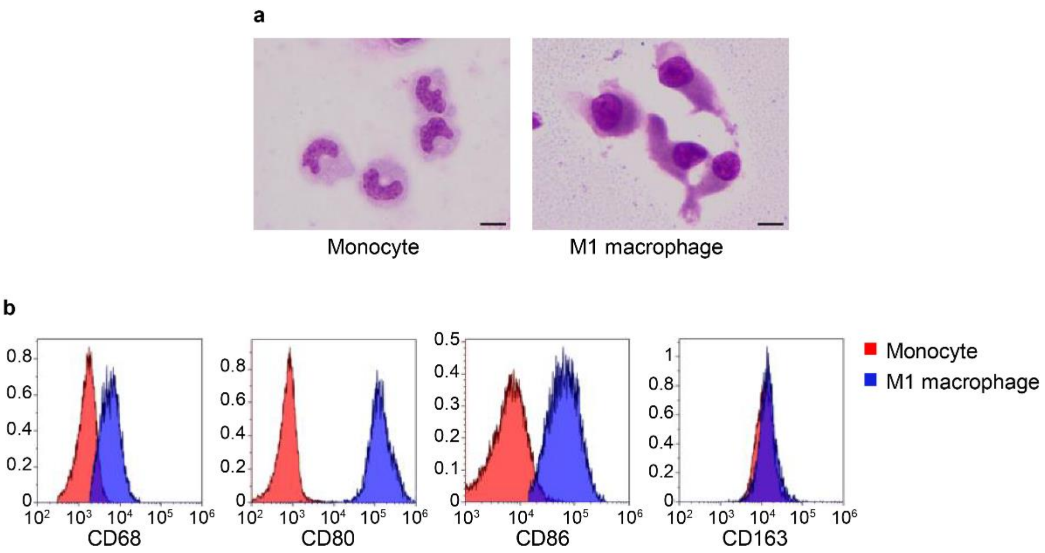

Figure S7. Monocytes isolated from the peripheral blood of healthy participants differentiated into M1 MΦs. (a) Cell morphology images of untreated monocytes and differentiated M1 MΦs (MG stain, magnification 1,000×). Scale bar: 10 µm. (b) Cell surface antigen analysis of M1 MΦs using flow cytometry.

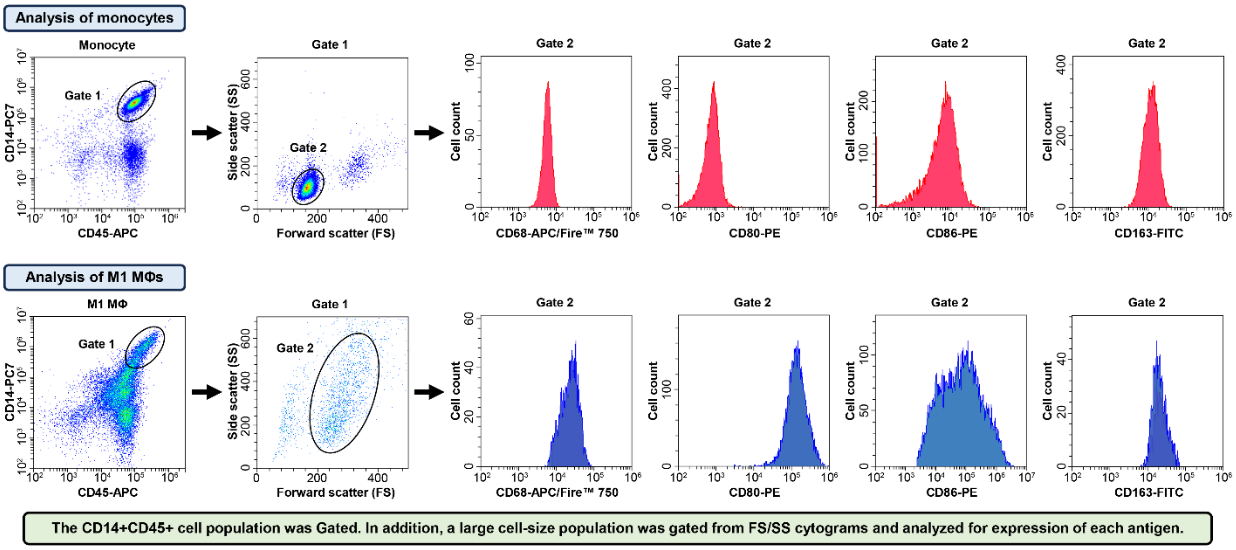

Figure S8. Gating strategy for analysis of cell surface antigens on monocytes and M1 MΦs using flow cytometry. Monocytes and M1 MΦs were collected and measured in a flow cytometer;

CD14$^+$ CD45$^+$ cell populations were gated, then large cell size populations were gated from FS/SS cytograms and analyzed for the expression of each antigen on monocytes and M1 MΦs.

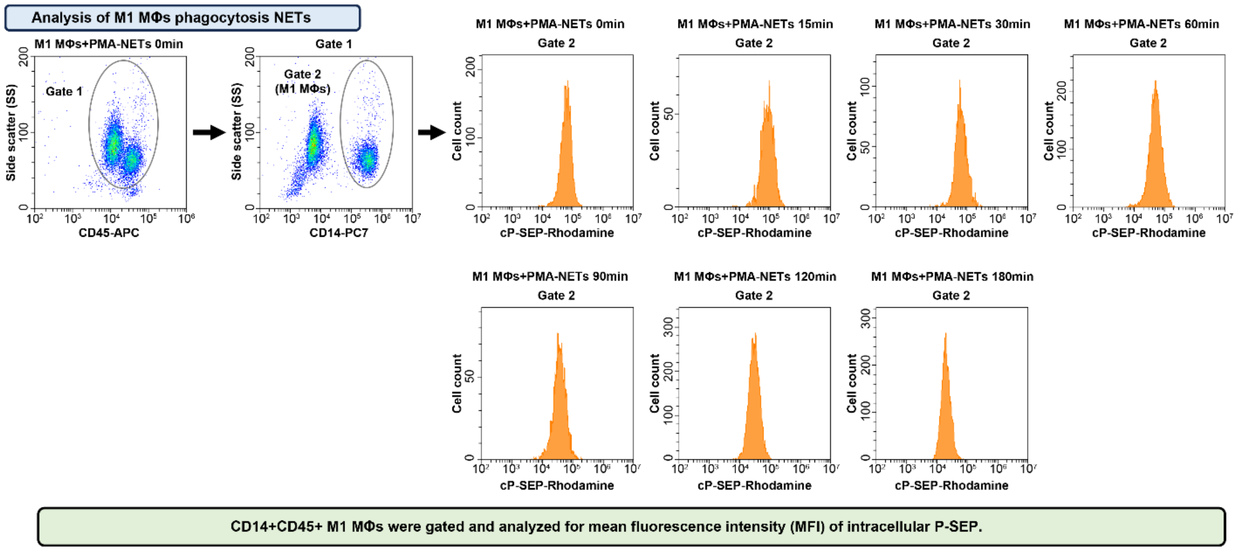

Figure S9. Gating strategy for analysis of NET phagocytosis M1 MΦ using flow cytometry. After co-culturing M1 MΦs and NETs, total cells were measured using flow cytometry; cell populations positive for CD45 and CD14 were gated and analyzed for P-SEP expression within M1 MΦs.

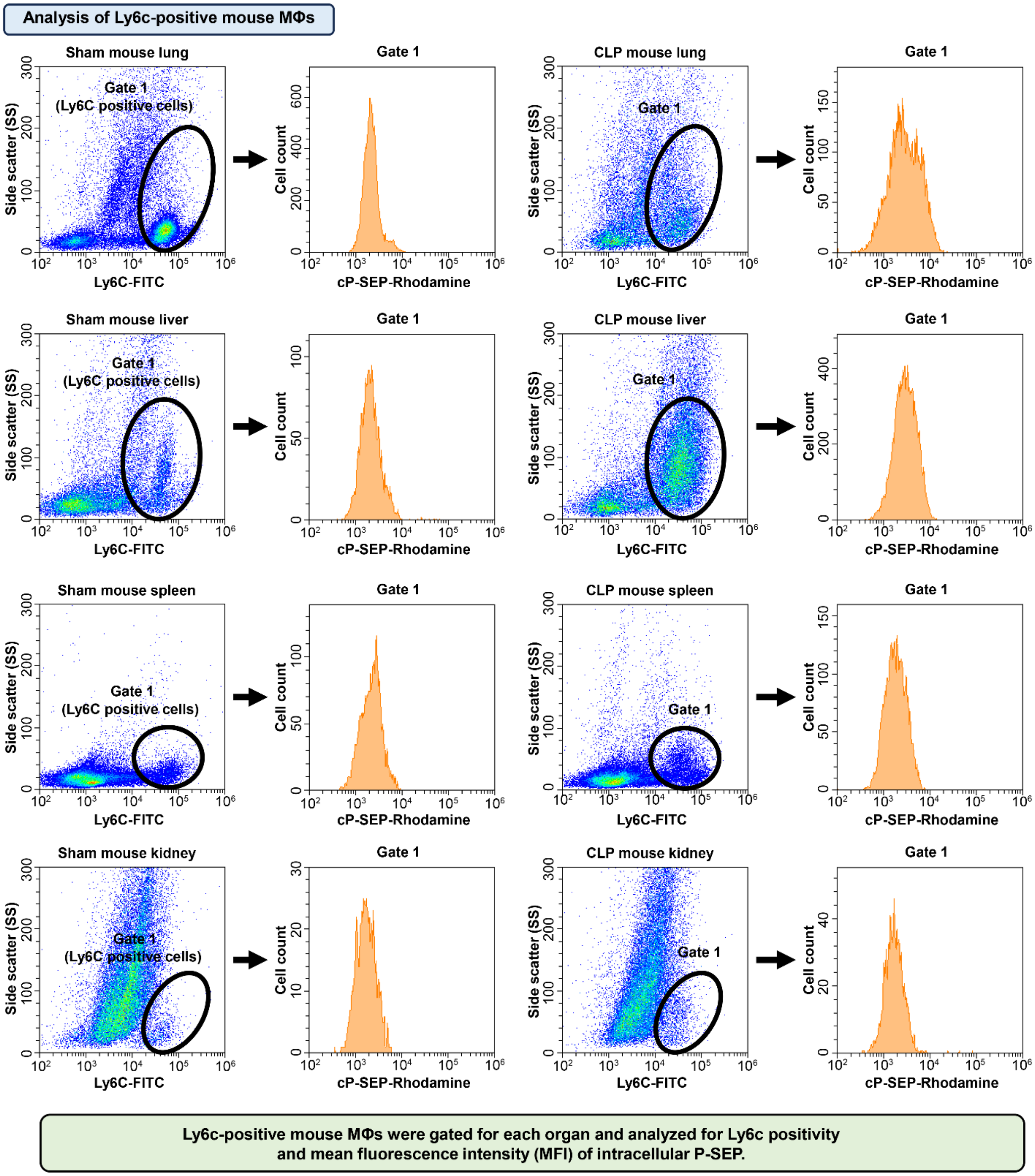

Figure S10. Gating strategies for analysis of mouse MΦ using flow cytometry. Cells collected from mouse organs were measured in a flow cytometer; Ly6C-positive MΦs were gated to analyze Ly6C positivity and P-SEP expression levels within mouse MΦs.

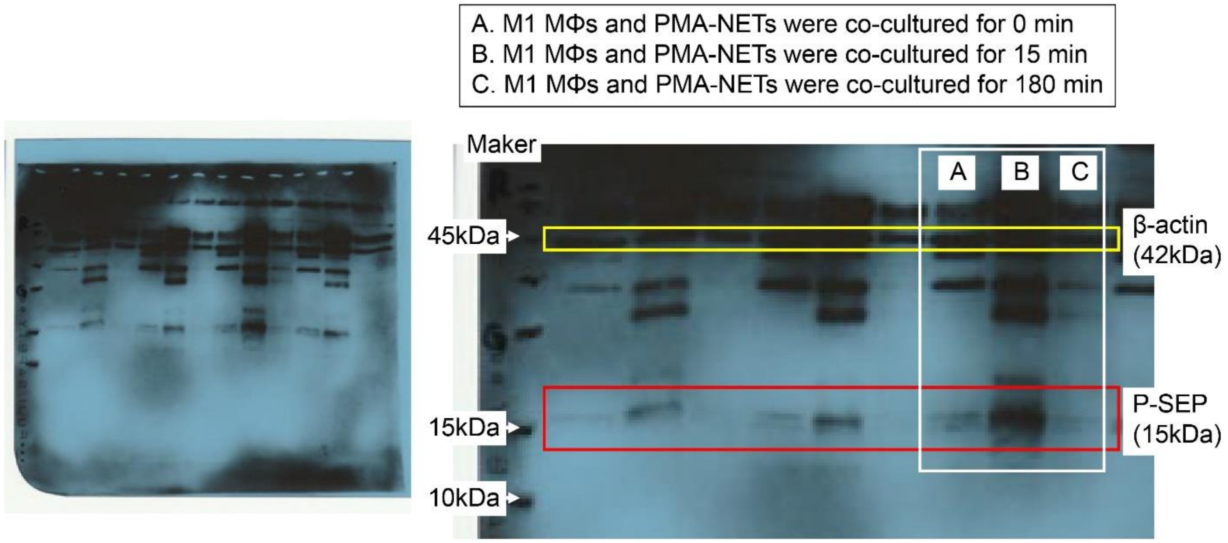

Figure S11. Unprocessed images of the western blot gels shown in Figure 1f. Western blotting was used to detect β-actin and P-SEP expression in samples at 0, 15, and 180 min after co-culture of M1 MΦ and PMA-NETs. Images within the white lines were used to produce Figure 1f.

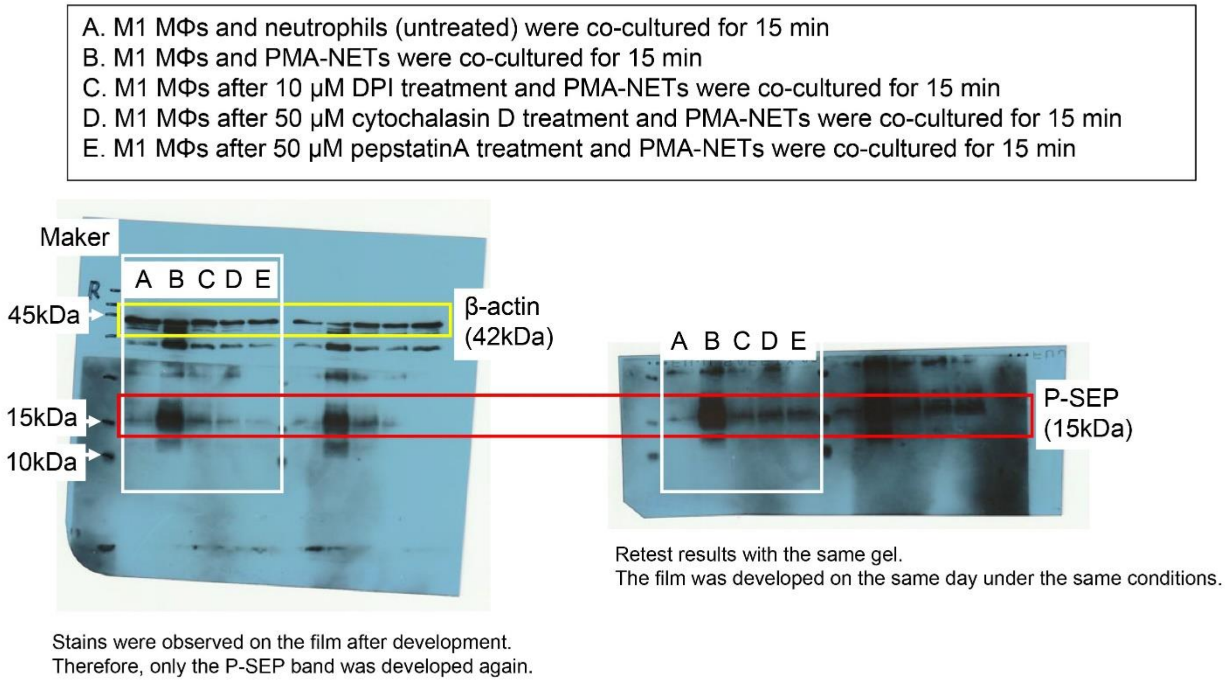

Figure S12. Unprocessed images of the western blot gels shown in Figure 3b. Western blotting was used to detect β-actin and P-SEP expression in samples co-cultured with M1 MΦ and

neutrophils, samples co-cultured with M1 MΦ and PMA-NETs, and samples after the addition of each inhibitor. Images within the white lines were used to produce Figure 3b.

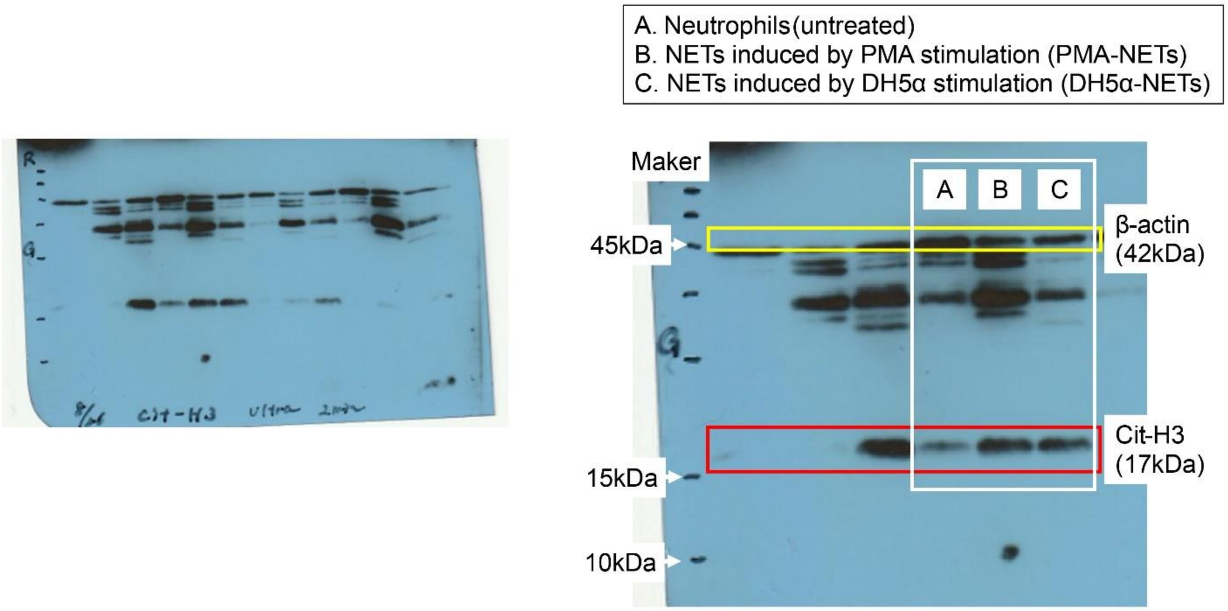

Figure S13. Unprocessed images of the western blot gels shown in Figure S2e. Western blotting was used to detect β-actin and Cit-H3 expression in samples unstimulated neutrophils (untreated), samples NETs induced by PMA and DH5α. Images within the white lines were used to produce Figure S2e.
